# From milling to digestion: matrix-driven effects of tritordeum and wheat flours on the technological quality and digestibility of cereal-based foods

**DOI:** 10.1016/j.crfs.2026.101595

**Published:** 2026-10-12

**Authors:** Giovanni D’Auria, Claudia Sardella, Andrea Bresciani, Felicia Graziuso, Chiara Nitride, Luisa Pellegrino, Pasquale Ferranti, Alessandra Marti, Massimo Blandino

**Affiliations:** a Department of Agriculture, University of Naples Federico II, Parco Gussone, Portici, Naples, 80055, Italy; b Department of Agricultural, Forest and Food Sciences, University of Turin, Largo P. Braccini 2, Grugliasco, Turin, 10095, Italy; c Department of Food, Environmental and Nutritional Sciences (DeFENS), University of Milan, Via G. Celoria 2, Milan, 20133, Italy

**Keywords:** *Triticum aestivum* L. ssp. *aestivum*, × *Tritordeum martinii*, Milling, *In vitro* digestion, Protein profiling, Bakery products, Fresh pasta

## Abstract

Commercial tritordeum and bread wheat grains have been roller- or stone-milled to produce refined and semi-whole flours for fresh pasta, breadsticks, bread, and pizza bases. The products were characterized to establish their technological properties and subjected to the standardized INFOGEST static *in vitro* digestion model. Despite its higher protein content and maximum GlutoPeak torque, tritordeum flour developed a less elastic and stable dough network, consistent with its lower relative abundance of HMW glutenin subunits. These characteristics limited gas retention and loaf expansion in the roller-milled bread. Pasta cooking loss did not differ between cereals within either milling method, whereas stone-milled tritordeum pasta showed greater firmness and work of shear than wheat counterpart. Tritordeum also yielded harder and tougher breadsticks and softer pizza bases than wheat with both milling methods. As no statistically significant differences were observed in the protein profiles of the roller- and stone-milled flours, protein and starch digestibility analyses were only carried out on roller-milled samples. The food matrix significantly affected protein digestibility, with bread and pizza exhibiting the highest values, regardless of the cereal type. The roller-milled tritordeum bread and fresh pasta exhibited significantly higher protein digestibility than the bread wheat counterparts, together with an apparent higher release of essential amino acids in the soluble intestinal fraction, thereby indicating differences in the digestive behavior of proteins in the two cereals. Furthermore, the roller-milled tritordeum bread showed a smaller proportion of digestible starch after prolonged digestion together with a higher resistant starch content, indicating differences in starch digestibility that may have implications for postprandial glycemia, which require confirmation *in vivo*. Overall, the results demonstrate that the flour composition, processing, and food matrix architecture jointly determined the technological performance, and they also provide new insights into the protein and starch digestibility of tritordeum-based foods.

## Highlights

• Tritordeum showed a higher number of bands in the 75-100 kDa region than wheat.
• Tritordeum developed a less elastic and stable dough network than wheat.
• The tritordeum bread and fresh pasta showed higher protein digestibility than wheat.
• The tritordeum bread had less digestible starch and more resistant starch.

## Graphical abstract

**Figure:**
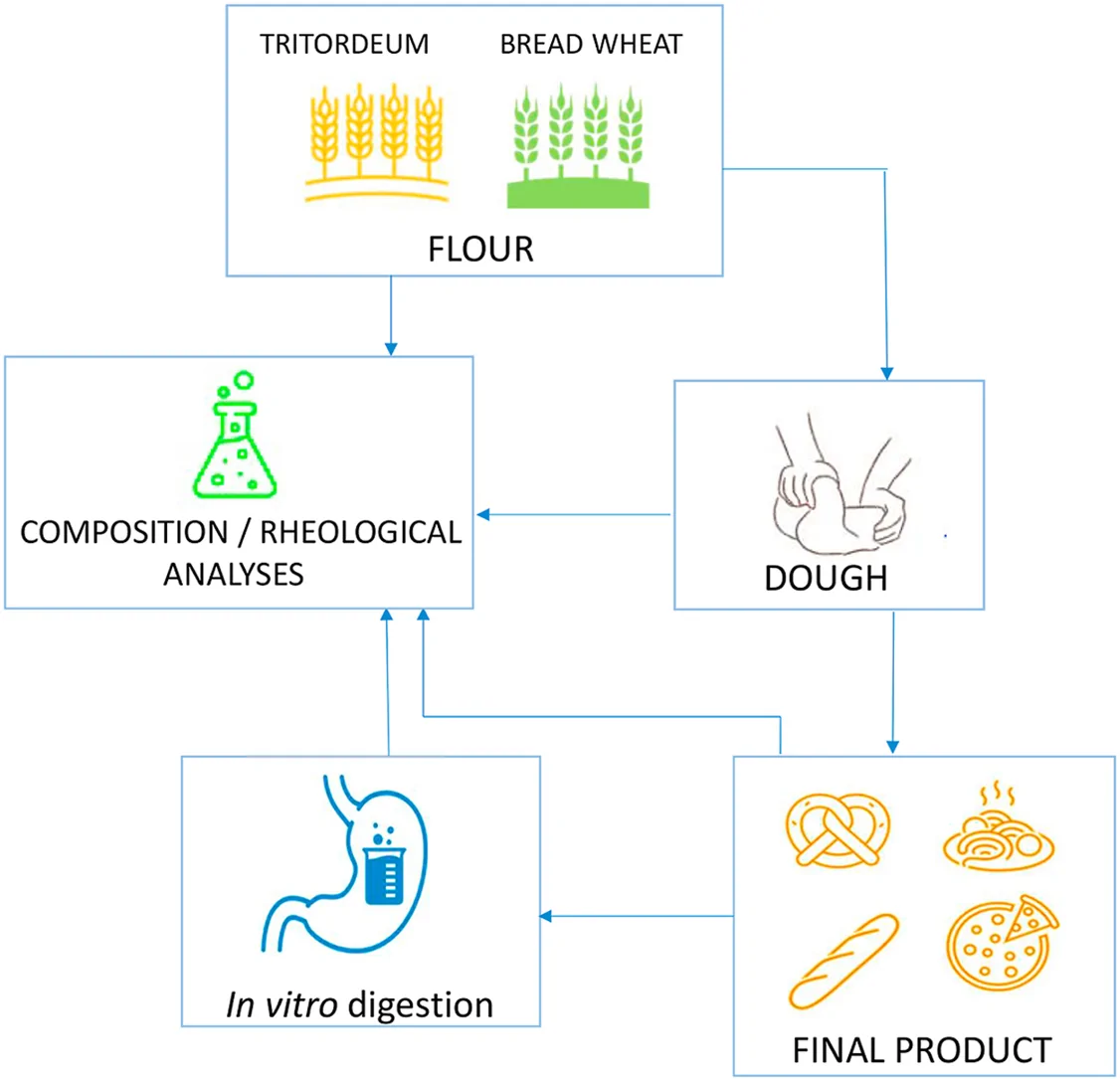

## 1 Introduction

Tritordeum (× *Tritordeum martinii* A. Pujadas, nothosp. nov.) is a novel cereal species that was commercially released in 2013 (CPVO, 2026). It was obtained from the hybridization of the *Hordeum chilense* Roem. et Schultz perennial wild barley species (genome H^ch^H^ch^, used as the female parent) with tetraploid durum wheat (*Triticum turgidum* ssp. *durum*, AABB) (Martín et al., 1999; Vaquero et al., 2018). Although durum wheat is the male parent of this hexaploid cereal (AABBH^ch^H^ch^), tritordeum resembles bread wheat in terms of grain texture, the rheological properties of the dough, and its baking quality (Martín et al., 1999). The grain texture of bread wheat is primarily determined by the expression of the *Pina-D1* and *Pinb-D1* genes, located on chromosome 5DS, which encode puroindoline A and B endosperm proteins (Yanaka et al., 2011). The functional alleles of both genes confer a soft kernel texture, whereas their absence or mutation results in hard grains. Quayson et al. (2018) suggested that puroindolines interact with hydrophobic regions of gluten proteins in grains or flour, alter the gluten protein organization, and facilitate protein solvation prior to dough mixing. Puroindoline wheat gene homologues (*Hina*-*H*^*ch*^*1* and *Hinb*-*H*^*ch*^*1*) on chromosome 5HS (Guzmán and Alvarez, 2014) may explain why the milling and rheological behaviors of tritordeum are more similar to those of bread wheat than durum wheat, which makes it more suitable for the production of bread and other leavened baked products than for pasta (Ávila et al., 2021).

There has been a recent increase in studies on tritordeum and the technological properties of its flour (Arora et al., 2022; Wiwart et al., 2025), particularly because of some compositional traits and related benefits that have been associated with its consumption. Tritordeum has been shown to exhibit three to five times more carotenoids than bread and durum wheat, a characteristic likely inherited from wild barley, with lutein being the predominant compound (Atienza et al., 2007; Sardella et al., 2026). This xanthophyll plays a key role in protecting the retina from photochemical damage (Stringham et al., 2010), and it contributes to the distinctive yellow color of tritordeum-based products (Ávila et al., 2021). The number of preserved carotenoids has been found to be higher in breadsticks, followed by bread, fresh pasta, and pizza, than the initial value of the flour (Sardella et al., 2026). The higher initial carotenoid content of tritordeum allows it to supply considerably more carotenoids than wheat to the end products, especially when refined flour is used instead of semi-whole flour (Sardella et al., 2026). Furthermore, although tritordeum is not suitable for individuals who suffer from celiac disease, lower levels of gluten immunogenic epitopes have been reported for its flour than for bread wheat, likely due to the absence of the D genome (Vaquero et al., 2018; Landolfi et al., 2021; Nitride et al., 2022), while a lower cell viability and monolayer permeability toxicity has been reported for tritordeum protein digested-samples than for durum wheat (De Caro et al., 2024). When used for *in vivo* studies, tritordeum was well tolerated by individuals with non-celiac wheat sensitivity (Sánchez‐León et al., 2021), and it ameliorated symptoms in patients with diarrhea-predominant irritable bowel syndrome (Russo et al., 2023).

Despite the increasing body of literature on the compositional and technological properties of tritordeum at the grain (Shewry et al., 2023; Giordano et al., 2019) and flour (Vaquero et al., 2018; Wiwart et al., 2025) levels, the relationship between its molecular and functional characteristics and its performance in different food matrices remains poorly understood. Moreover, although other studies have focused on bread models (Vaquero et al., 2018; Nitride et al., 2022; Sánchez‐León et al., 2021), the impact of the milling and processing conditions on the structure of tritordeum-based products and their effect on digestive behavior has not been investigated. However, it has been established that the food matrix plays a crucial role in modulating macronutrient bioaccessibility and digestion kinetics (Ferrara et al., 2025). Thus, a deeper understanding of how the flour refinement level, protein organization, and processing conditions interact in determining both the technological performance and digestive outcomes is essential to fully exploit the potential of this cereal in food applications.

The objectives of the present study have been (1) to assess the impact of roller and stone milling on flour composition and on gluten and starch rheological properties of one tritordeum variety and one of bread wheat; (2) to relate these characteristics with the suitability of using tritordeum in different food applications, i.e., fresh pasta, breadsticks, bread, and pizza bases; and (3) to elucidate how the flour properties and food structure influence the macromolecular breakdown and the metabolic fate of the digestion products. This work provides new insights into the effect of the matrix on protein and starch digestibility, thereby providing a scientific basis for the use of tritordeum on the global food market.

## 2 Materials and methods

### 2.1 Plant material and milling processes

Overall, 100 t grains of a tritordeum variety (cv. Bulel, Arcadia S.p.A., Pamplona, Spain) and 100 t of a bread wheat variety (cv. Graindor, Unisigma, Froissy, France) were ground by either roller milling (GBS S.n.c., Leinì, Italy) or stone milling (Partisani S.r.l., Forlì, Italy) in an industrial plant (Molini Bongiovanni S.p.A., Cambiano, Italy) to obtain refined and semi-whole flours, respectively, as described in Sardella et al. (2026). Briefly, samples were collected from the opening slits of the milling plant, according to the sampling method reported in European Commission Regulation (EC) No. 401/2006 (European Commission, 2006). An aggregate representative sample was obtained for each lot and milling process and stored at +4 °C until being processed. Roller milling allowed the bran (milling yield 8%), shorts (9%), and red dog (5%) to be separated from the refined flour (78%). A further sifting process was performed after stone milling, using a single channel square purifier (GBS S.n.c.), to separate 10% of bran and brown flour from the semi-whole flour (90%). The particle size of the refined and semi-whole flour was measured using a set of 6 sieves (Retsch, Haan, Germany) with progressively smaller mesh sizes, as reported in Table S1.

### 2.2 Flour composition and rheological properties

The protein and gluten contents of the flours were determined according to AACC method 39-10.01 (AACC, 2008) by means of XDS NIR (XM-1100 Series, FOSS Italia S.r.l., Padua, Italy). The ash content was measured according to the muffle furnace (AOAC 923.03) method (AOAC, 2005). The monosaccharide composition of the arabinoxylans was determined by high-performance liquid chromatography with reflectance index detection (HPLC-RID), as previously reported (Sardella et al., 2023). The flours were tested for the Hagberg falling number using a Perten Instruments Falling Number 1500 machine (Milan, Italy) according to ISO 3039. The moisture content was determined by oven-drying (Falc Instrument S.r.l., Treviglio, Italy) the flours at 105 °C to a constant weight to calculate the dry weight (dw). All the analyses were performed in triplicate.

Damaged starch was assessed on the raw materials, according to the standard method AACC 76-31.01 (AACC, 2008). The damaged starch content was expressed as the total amount of starch, according to the standard method AACC 76-13.01 (AACC, 2008). Both analyses were performed in triplicate, and the average value was reported. The starch pasting properties were evaluated on raw materials, using a Brabender® Micro-Visco-Amylograph (Anton Paar Italia, Rivoli, Italy). An aliquot of 12 g of sample was dispersed in 100 mL of distilled water, and both the flour and water weights were scaled on a 14% flour moisture basis. The suspensions were subjected to the following standard temperature profile: heating from 30 °C to 95 °C, holding at 95 °C for 20 min, and cooling from 95 °C to 30 °C, and they were stirred at 250 min^−1^ using a 300 cm•gf cartridge. A heating/cooling rate of 3 °C min^−1^ was applied. The gluten aggregation properties were measured in triplicate using a Brabender® GlutoPeak device (Anton Paar Italia). Specifically, 9 g of the samples were added to 9 ml of distilled water and mixed at 2750 rpm at 35 °C. The mixing properties were assessed in duplicate, using a Brabender® Farinograph-E (Anton Paar Italia) equipped with a 300 g mixing bowl, following the ICC 115/1 Approved Method (ICC, 1992). Finally, the extensional properties were evaluated in duplicate, by means of a Chopin® Alveograph (KPM Analytics, MA, the USA), according to the standard method AACC 54-30.02 (AACC, 2008).

### 2.3 Technological processes

The refined and semi-whole flours of both tritordeum and wheat were used to prepare fresh pasta, breadsticks, bread, and pizza bases. Three trials were performed for each product formulation, and three representative samples were collected for each trial, which included a combination of species (tritordeum vs bread wheat), milling degrees (78% vs 90% extraction rates), and processing types. The kernels, the refined and semi-whole flours, and the developed products are presented in Fig. S1. The products were selected because they have similar formulations but different processing procedures, i.e. specific technological parameters in the kneading, leavening, and cooking/baking stages, as described in a previous study by Sardella et al. (2026) and summarized in the experimental workflow of Fig. S2. For bread and pizza bases, water addition was initially based on farinograph water absorption at 500 FU and was subsequently increased empirically during the production trials. For breadsticks and fresh pasta, water addition was determined empirically by the operators conducting the respective production trials.

An aliquot of 2 kg of flour was mixed with water (40 °C), at different ratios, depending on the farinograph water absorption (roller-milled tritordeum 46.5% and wheat 40.0%, stone-milled tritordeum 50.0% and wheat 44.1%) to prepare fresh pasta, and the doughs were processed in a planetary kneading machine (Kenwood, De’ Longhi Appliances S.r.l., Treviso, Italy) for 6 min at 25 °C, left to rest for 30 min at 4 °C, then rolled into 1 mm thick pasta sheets using a continuous press (Imperia & Monferrina S.p.a., Moncalieri, Italy) and shaped into 10 × 10 cm squares. The pasta was cooked in boiling distilled water (pasta:water ratio = 1:10) for the optimum period of time, which had been determined by ten individuals through a sensory evaluation of the products (1.5 min for the roller-milled samples and 1.0 min for the stone-milled samples). Specifically, starting at 1 min of cooking, pasta strands were sampled at 30-s intervals and tasted. Each assessor independently identified the optimum cooking time based on perceived firmness and structural integrity. The final cooking time for each sample was calculated as the mean of the times selected by the assessors.

Breadsticks were produced at an industrial scale in a local factory (Giovanni Cane S.r.l., Alessandria, Italy). The flours were mixed in a kneading machine (Sancassiano S.p.A., Roddi, Italy) for 10 min at 29 °C with corn oil (6% on a flour basis), fresh yeast (5%), salt (1.7%), and water in different ratios, depending on the type of flour (roller-milled tritordeum 27.9% and wheat 26.7%, stone-milled tritordeum 29.6% and wheat 27.5%). The dough was left to rest (20 min, 27 °C, 45% relative humidity, RH) and then sheeted and folded for about 10 min. After leavening for 35 min (36 °C, 75% RH), the dough was shaped, using stainless steel molds (Torneria Automatica Tealdi S.n.c., Clavesana, Italy), and baked in a continuous tunnel oven (Tibiletti Forni S.r.l., Novara, Italy) for 8.5 min at 280 °C.

An aliquot of 3 kg of flour was mixed with fresh yeast (3% on the flour basis), salt (2.5%), and water in different ratios, depending on the type of flour (roller-milled tritordeum 60.3% and wheat 53.8%, stone-milled tritordeum 66.6% and wheat 58.4%) to prepare bread, and then processed in a spiral kneading machine (Esmach Ali Group S.r.l., Grisignano di Zocco, Italy) for 11 min at 25 °C. The dough was left to rest for 30 min at 30 °C and 70% RH, and then manually divided into 750 g loaves, which, after leavening for 60 min at 30 °C and 70% RH, were baked in a bakery oven (Moretti Forni S.p.A., Mondolfo, Italy) for 40 min at 220 °C.

An aliquot of 3 kg of flour was mixed with fresh yeast (1% on a flour basis), salt (2.5%), and water in different ratios, depending on the type of flour (roller milled-tritordeum 60.3% and wheat 53.8%, stone-milled tritordeum 66.6% and wheat 58.4%) to prepare pizza bases, and processed in a spiral kneading machine (Esmach Ali Group S.r.l.) for 13 min at 25 °C. The dough was left to rest for 30 min at 24 °C and 60% RH, and then manually divided into 300 g loaves. After leavening for 17 h at 4 °C, the loaves were kneaded by hand and left to rest for another 2 h (24 °C, 60% RH). The loaves were then shaped and baked in a bakery oven (Moretti Forni S.p.A.) for 5 min at 320 °C, without the addition of any ingredients as toppings.

### 2.4 Characterization of the products

#### 2.4.1 Chromatic characteristics of the flours and derived products

The color of both the raw materials and products was determined using a reflectance colorimeter (CR 210, Minolta Co., Osaka, Japan) to measure the lightness and saturation of the color intensity. The results were expressed in the CIE L* a* b* color space. Measurements were performed in triplicate.

#### 2.4.2 Specific volume and texture of the baked products

The specific volume of the bread was evaluated considering the ratio between the volume, which was calculated according to international method AACC 10-05.01 (AACC, 2008), and the mass. Bread and pizza firmness was assessed, using a texture analyzer (TA.XT-plus, Stable Micro Systems Ltd., Godalming, the UK) equipped with a 100 N load cell and a 36 mm probe (Radiused Cylinder Probe-P36R). The AACC 74-09.01 (AACC, 2008) method was used at a test speed of 100 mm min^−1^.

Breadstick firmness was assessed, using a texture analyzer (TA.XT-plus, Stable Micro Systems Ltd.) equipped with a 100 N load cell. Samples were compressed, using a HDP/3 PB probe at a crosshead speed of 1 mm s^−1^ for 3 mm. Six replicates were carried out for both the bread and pizza bases, whereas ten replicates were considered for the breadsticks.

#### 2.4.3 Characterization of the fresh pasta

Fresh pasta was cooked in distilled water (pasta:water ratio = 1:10) at the optimum cooking time. After cooking, the pasta was drained for 30 s, and the water absorption of the pasta was determined gravimetrically. The cooking water was collected to evaluate the cooking losses (g of solid loss per 100 g of pasta), according to AACC 66-52.01 (AACC, 2008). Finally, the firmness of the cooked pasta was evaluated, on 5 “tagliatelle”, using a TA.XT-plus Texture Analyzer (Stable Micro Systems Ltd.), equipped with a light knife blade (A/LKB-F) and a 100 N load cell (crosshead speed of 10.0 mm min^−1^), according to AACC 66-52.01 (AACC, 2008). Pasta firmness was assessed directly after a 30 s drain phase. Water absorption, cooking loss, and pasta firmness were analyzed in triplicate in three independent cooking trials.

### 2.5 Analytical determinations

#### 2.5.1 Sample preparation

Three representative flour and dough samples were collected separately for each treatment and immediately stored at +4 °C for analytical determination. The doughs, collected just before the cooking/baking step, were freeze-dried, using a Lyovapor L-200 Freeze Dryer (Buchi, Flawil, Switzerland), ground to a fine powder (particle size < 500 μm) with a Cyclotec 1093 sample mill (Foss, Padua, Italy) and stored at −25 °C until analysis. Baked products and freshly cooked pasta were immediately ground and homogenized with a cutting mill after production. The homogenized material was subsequently used for *in vitro* digestion and protein extraction for the protein profiling analyses.

#### 2.5.2 Chemicals

All the reagents and solvents used in this study were of analytical or higher grade, and they were purchased from Carlo Erba Reagents S.r.l. (Milan, Italy). The chemicals used to produce the simulated digestive fluids were provided by Carlo Erba Reagents S.r.l. The enzymes used for *in vitro* human digestion were purchased from Sigma-Aldrich (St Louis, MO, the USA), in line with those recommended by the INFOGEST protocol (Brodkorb et al., 2019). Trichloroacetic acid (TCA), sodium dodecyl sulfate, glycerol, tris(hydroxymethyl)aminomethane hydrochloride (Tris-HCl), trifluoroacetic acid, acetonitrile, and ρ-toluene-sulfonyl-L-arginine methyl ester (TAME) were also acquired from Sigma-Aldrich. The electrophoresis reagents were all obtained from Bio-Rad (Milan, Italy). The protein-free bread (“Amino Pane Le Rosette”, produced by Antica Farmacia Orlandi, with a protein content < 0.1%, w/w), was purchased from a local pharmacy.

#### 2.5.3 Selective extraction of gliadin and glutenin

A selective fractionation protocol was employed to isolate the gliadin and glutenin fractions from the flours (both roller and stone milled) in order to characterize the storage protein profile, according to established methods (Landolfi et al., 2021; Nitride et al., 2022). The gliadin and glutenin extracts were precipitated with an equal volume of 40% TCA for 1 h at 25 °C in an orbital shaker to calculate the gliadin-to-glutenin (GLI/GLU) ratio. The TCA samples were centrifuged for 15 min at 12000 × *g* at 4 °C to recover the precipitated protein fractions. After centrifugation, the residual TCA was removed by washing the pellet three times with cold acetone (−18 °C). The precipitated proteins were then resuspended in a denaturing and reducing buffer (3 M urea, 50 mM ammonium hydrogen carbonate, and 5 mM dithiothreitol).

#### 2.5.4 Protein extraction from the doughs and products

The total proteins were isolated from both the lyophilized doughs and the finished products, according to the procedure described by Korte et al. (2019), with minor modifications, to maximize the protein yield. Briefly, 1 g of homogenized matrix was dissolved in 10 mL of 6 M urea, 1 M thiourea, and 50 mM Tris buffer, pH 8.0 (1:10, w:v). The protein extraction was performed at room temperature (20 °C) for 1 h on a rocking plate.

#### 2.5.5 Protein quantification of the flour protein fractions, doughs, and food products

The protein concentration of the selectively extracted flour protein fractions was determined using the Bradford assay (Cod. B6916, Sigma-Aldrich). Protein extracts were mixed with Bradford reagent at a 1:30 (v/v) ratio and analyzed according to the manufacturer's instructions. A calibration curve was prepared using bovine serum albumin as the external standard (0.2–1 mg mL^−1^). After 5 min of incubation, the protein concentration was determined spectrophotometrically at λ = 595 nm. Data were adjusted using a conversion factor of 4.25 for bovine serum albumin/gliadin and 3.42 for bovine serum albumin/glutenin, as previously reported (Rekowski et al., 2021). The protein concentration of all the protein solutions containing a high concentration of urea (6 M) was determined spectrophotometrically according to D'Auria et al. (2025).

#### 2.5.6 SDS-PAGE analysis

Protein extracts were mixed 3:1 (v/v) with a reducing Laemmli buffer 4X [0.2 M Tris-HCl, pH 6.8, containing 8% (w/v) SDS, 40% (w/v) glycerol, 100 mM DTT and traces of bromophenol blue], boiled for 10 min in a water bath and centrifuged at 7900 × *g* for 5 min (Microcentrifuge Multispin 12, Steroglass, Perugia, Italy) for SDS-PAGE (sodium dodecyl sulfate-polyacrylamide gel electrophoresis) analysis. An aliquot of 15 μg of protein was loaded onto the wells of a 7.5–13% gradient polyacrylamide gel (Singh et al., 1991). Precision Plus Protein™ Standards (Bio-Rad Laboratories, Inc., Hercules, Ca, the USA) were used as the molecular marker. The proteins were separated at room temperature (20 °C) and at a constant voltage (120 V). Gels were fixed in 24% (w/v) TCA, left overnight (16 h), and rinsed three times in Milli-Q water by gently shaking for 5 min. The gels were stained for 16 h in QC Colloidal Coomassie Stain G-250 (Bio-Rad Laboratories, Inc.) de-stained in Milli-Q water and left overnight (16 h). To assess potential changes in the protein profile associated with dough preparation and cooking (i.e., the presence of inter- and intramolecular adducts), the protein profiles of the doughs and corresponding cooked products were analyzed under reducing (0.05 M DTT) and non-reducing (no DTT) conditions (Fig. 2). The gels were imaged and processed using GelAnalyzer, version 23.1 [GelAnalyzer 23.1 (www.gelanalyzer.com) by Istvan Lazar Jr., PhD and Istvan Lazar Sr., PhD, CSc]. The apparent molecular mobility of the unknown proteins was estimated, with a cubic spline curve, using the SRS1 Cubic Spline for Excel software (SRS1 Software, 2026).

#### 2.5.7 Reversed-phase (RP)-HPLC analysis

RP-HPLC analysis of the gliadins and glutenins was performed, using an Agilent HP 1100 modular system (Palo Alto, CA, the USA) equipped with a diode array detector. The column effluent was monitored via UV detection at λ = 220 nm. Separation was carried out on a C4 Vydac 214TP52 column (2.1 × 250 mm; Hesperia, Ca, the USA). Solvent A comprised 0.1% trifluoroacetic acid in water, while solvent B included 0.07% trifluoroacetic acid in acetonitrile. The flow rate was set at 0.200 mL min^−1^, using the following solvent B gradient: 25% at 0 min, 30% at 5 min, 60% at 65 min, and 100% at 70 min. The peak area was integrated, using Agilent ChemStation software, to obtain the relative quantities of both the gliadins and glutenins in each sample.

### 2.6 In vitro static simulated digestion of fresh pasta and baked products

Digestion was performed on freshly prepared products obtained only from roller milling, according to the standard cooking procedure described in Paragraph 2.3. Simulated gastrointestinal digestion, including oral, gastric, and duodenal phases, was performed using the standardized INFOGEST protocol (Brodkorb et al., 2019). The enzymatic potency of pancreatin was verified by monitoring the hydrolysis of TAME at 247 nm for 10 min (Brodkorb et al., 2019). The specific trypsin activity was determined to be 9.5 U mg^−1^ of pancreatin powder. After duodenal digestion, samples were boiled for 5 min, to interrupt enzymatic digestion, and then centrifuged at 3433 × *g* for 30 min (Centrifuge XS R-8D, Remi Instruments, New Delhi, India) at room temperature (+20 °C). The obtained supernatant, containing the soluble fraction of the digestion products, was used for the subsequent analysis, while the pellet represented the fraction that remained undigested. Only the duodenal digest at 120 min was used for protein digestibility studies. A blank (protein-free) control was subjected to the same digestion conditions as the experimental food samples to account for the contribution of protein autolysis. Starch digestibility was studied in the bread samples, and the duodenal phase was prolonged for 300 min. Aliquots of the duodenal digests of bread were collected at 0, 20, 40, 60, 90, 120, 150, 180, 240 and 300 min, and then boiled and centrifuged as previously described. All digestions were performed in technical duplicates.

#### 2.6.1 Acidic hydrolysis of the duodenal digesta and products and determination of the degree of protein hydrolysis

The samples used for the determination of the protein digestibility parameters were prepared as previously described (Ferrara et al., 2025). Briefly, the soluble duodenal fraction, containing free amino acids and soluble peptide fragments, was subjected to acid hydrolysis prior to amino acid analysis and determination of the degree of protein hydrolysis. Afterwards, hydrolyzed samples were cleaned, by centrifugation at 7900 × *g* for 5 min, diluted with an equal volume of 6 N NaOH, adjusting the pH to 7.0 ± 0.5, and the protein hydrolysis degree (referred as total protein digestibility in Fig. 3a), was determined according to Ferrara et al. (2025). All the samples were assayed in technical duplicates.

#### 2.6.2 Ion-Exchange (IE)-HPLC analysis

The 120-min duodenal supernatant was analyzed to characterize the amino acid released during simulated digestion. A 0.2 mL volume of filtrate was dried, under vacuum, using a Rotavapor R-300 (Buchi, Milan, Italy) and redissolved with 50 μL of 0.2 N lithium citrate buffer, pH 2.2. The solution was filtered through a 0.2 μm membrane filter (Millipore, Milford MA, the USA) and analyzed, by means of ion exchange chromatography, using a Biochrom 30+ amino acid analyzer (Erreci, Milan, Italy) (Hogenboom et al., 2017). The individual amino acids content was calculated using six-level calibration curves. Considering the hydrolysis conditions adopted in the present study, the tryptophan and sulfur-containing amino acid contents were not determined. Asparagine and glutamine were converted into aspartic acid and glutamic acid, respectively. IE-HPLC analyses were performed as single measurements on a pooled digest.

#### 2.6.3 Starch digestion of the roller-milled bread

Starch digestion was evaluated by quantifying free D-glucose released during duodenal digestion (free glucose-derived starch – FGS) and the residual soluble starch-derived carbohydrate fraction after digestion (residual hydrolysable starch – RHS), using the K-DSTRS Megazyme protocol (Megazyme Ltd., Wicklow, Ireland) with some modifications (Englyst et al., 1996; Rotondi Aufiero et al., 2022).

Free D-glucose was quantified directly in the soluble digest at various timepoints, after a further centrifugation (7900 × *g* for 5 min), using an Enzytec™ Liquid D-Glucose kit (E8140, R-Biopharm AG), according to the manufacturer's instructions. Analyses were performed using an iCubio i-Magic M9 (R-Biopharm Italia S.R.L.). For the determination of the RHS, an aliquot of the soluble duodenal digesta was mixed in a 1:2 ratio (v/v) with amyloglucosidase (AMG) (from *Aspergillus niger*, 260 U mL^−1^, Ref: A7095, Merck, Darmstadt, Germany). The mixture was stirred for 30 min at 50 °C and centrifuged at 7900 × *g* for 5 min. The supernatant was analyzed for free D-glucose as described above. For samples collected after 300 min of duodenal digestion, the FGS was subtracted from the total starch equivalents determined after amyloglucosidase treatment, thereby estimating the RHS like poly-/oligosaccharides:

(1) RHS=totalstarchequivalentafterAMGtreatment−FGS

The glucose values were converted into starch equivalents using a glucose-to-starch conversion factor of 0.9 (Bai et al., 2021). An aliquot of 100 mg of the pellet, obtained from the 300-min duodenal sample, was washed twice with 50% ethanol (v/v) to remove residual malto-oligosaccharide and free glucose prior to resistant starch (RS) determination. After centrifugation, the pellet was suspended in a 1:20 (w/v) ratio in 1.7 M NaOH and stirred on a magnetic plate for 20 min. The suspension was then diluted 1:10 (v/v) with 1 N sodium acetate (pH 3.8). Subsequently, 600 μL of the samples was mixed with 90 μL of amyloglucosidase (260 U mL^−1^), and the reaction was carried out in a water bath at 50 °C for 30 min with intermittent mixing. The samples were centrifuged at 7900 × *g* for 5 min, and the supernatant was transferred into clean vials for glucose determination. The glucose generated during the reaction was quantified as described above and converted into starch equivalents using a glucose-to-starch conversion factor of 0.9 (Bai et al., 2021). RS was calculated based on the amount of starch equivalent recovered from the pellet and expressed as g 100 g^−1^ bread. All biological duplicate samples were assayed in technical duplicates, and the absorbance values were averaged. The reaction blank was used to correct for glucose arising from the analytical reaction/reagents; no correction for the endogenous free glucose content of the undigested bread was performed.

### 2.7 Statistical analysis

One-way analysis of variance (ANOVA) was conducted for each type of product (flours and the derived products) to compare the combination (*n* = 4) of monovarietal commercial lots (one variety of tritordeum vs one of bread wheat) and milling process (roller vs stone milling) on the chemical, biochemical, rheological, and quality traits of the flours and final products. Protein profiling of the flours was performed on both roller- and stone-milled samples. As no statistically significant differences were observed in the protein profiles between the two milling systems (Table 1), the subsequent protein profiling and digestibility experiments were restricted exclusively to doughs and products prepared from roller-milled flours. Therefore, milling was not considered as an independent experimental factor. Means were identified as significantly different (*p*-value < 0.05) on the basis of the Ryan-Einot-Gabriel-Welsch F (REGW-F) statistical test. A graphical method was used, prior to the analysis, to verify the basic assumptions. The analyses were carried out in the R environment (R version 4.4.2).

**Table 1:** Chemical, biochemical, and colorimetric characterization of the tritordeum and bread wheat flours obtained from roller and stone milling.

Parameter	Roller milling				Stone milling			
	Tritordeum		Bread wheat		Tritordeum		Bread wheat	
Protein content (% dw)	17.5 ± 0.1	a	12.7 ± 0.1	c	17.2 ± 0.2	a	13.7 ± 0.1	b
Gluten content (% dw)	39.6 ± 0.2	a	30.0 ± 0.2	c	33.2 ± 0.2	b	29.2 ± 0.2	d
Ash content (% dw)	0.89 ± 0.03	c	0.67 ± 0.01	d	1.23 ± 0.01	a	1.12 ± 0.01	b
Total arabinoxylans (% dw)	1.98 ± 0.30	bc	1.61 ± 0.12	c	3.07 ± 0.25	a	2.46 ± 0.22	b
HMW/LMW-GS	0.49 ± 0.01	b	0.72 ± 0.01	a	0.47 ± 0.01	b	0.73 ± 0.03	a
GLI/GLU	1.67 ± 0.34	a	1.46 ± 0.35	a	1.29 ± 0.12	a	2.32 ± 0.78	a
Hagberg falling number (s)	563.0 ± 2.0	a	372.3 ± 1.2	d	504.7 ± 3.2	b	399.3 ± 1.5	c

L*	90.9 ± 0.4	ab	92.4 ± 1.2	a	89.0 ± 0.5	b	89.2 ± 1.2	b
a*	0.68 ± 0.05	b	0.61 ± 0.04	b	1.80 ± 0.19	a	1.58 ± 0.12	a
b*	19.0 ± 0.3	a	12.2 ± 0.2	c	16.8 ± 0.4	b	13.0 ± 0.5	c

HMW-GS*,* high-molecular-weight glutenin subunits; LMW-GS*,* low-molecular-weight glutenin subunits; GLI, gliadins; GLU, glutenins; L* = luminosity; a* = redness; b* = yellowness. Means followed by different letters in the same row are significantly different, according to the REGW-F test (*p-*value < 0.05).

## 3 Results and discussion

### 3.1 Protein profiling of the flours

The electrophoretic profiles of the glutenin fractions (Fig. 1a) in the roller-milled samples showed the protein storage pattern of the wheat, with bands attributable to high-molecular-weight glutenin subunits (HMW-GS), mainly in the 75-100 kDa region, and a complex set of low-molecular-weight glutenin subunits (LMW-GS), distributed approximately between 30 and 60 kDa. The two varieties exhibited different qualitative band patterns, with tritordeum variety showing a higher number of bands (n = 7) than that of bread wheat (n = 5), thus suggesting a different expression of glutenin-related proteins. The gliadin proteome exhibited clear differences over the electrophoretic profile. This is in agreement with Nitride et al. (2022), who showed that tritordeum exhibited a distinct banding pattern, compared with both bread and durum wheat, particularly in the HMW-region (Mr > 50 kDa), where ω-gliadins typically migrate. This feature can be attributed to the presence of hordein proteins derived from the *H. chilense* genome, including D-hordeins (Mr > 60 kDa), C-hordeins (Mr > 40 kDa), and B-hordeins (Mr > 30 kDa), which are co-extracted with gliadins, because of their similar solubility in 70% (v/v) ethanol (Nitride et al., 2022; Yalçın and Çelik, 2007). From a functional perspective, these hordein fractions may contribute by altering the balance between the monomeric and polymeric protein components, thereby affecting the viscoelastic behavior of the gluten network. C-hordeins, being homologous to ω-gliadins, lack cysteine residues and therefore occur only in monomeric form, without participating in disulfide bond formation. Their relative abundance may influence the viscosity of dough and weaken intermolecular connectivity within the gluten network (Balakireva and Zamyatnin, 2016).

**Fig. 1:**
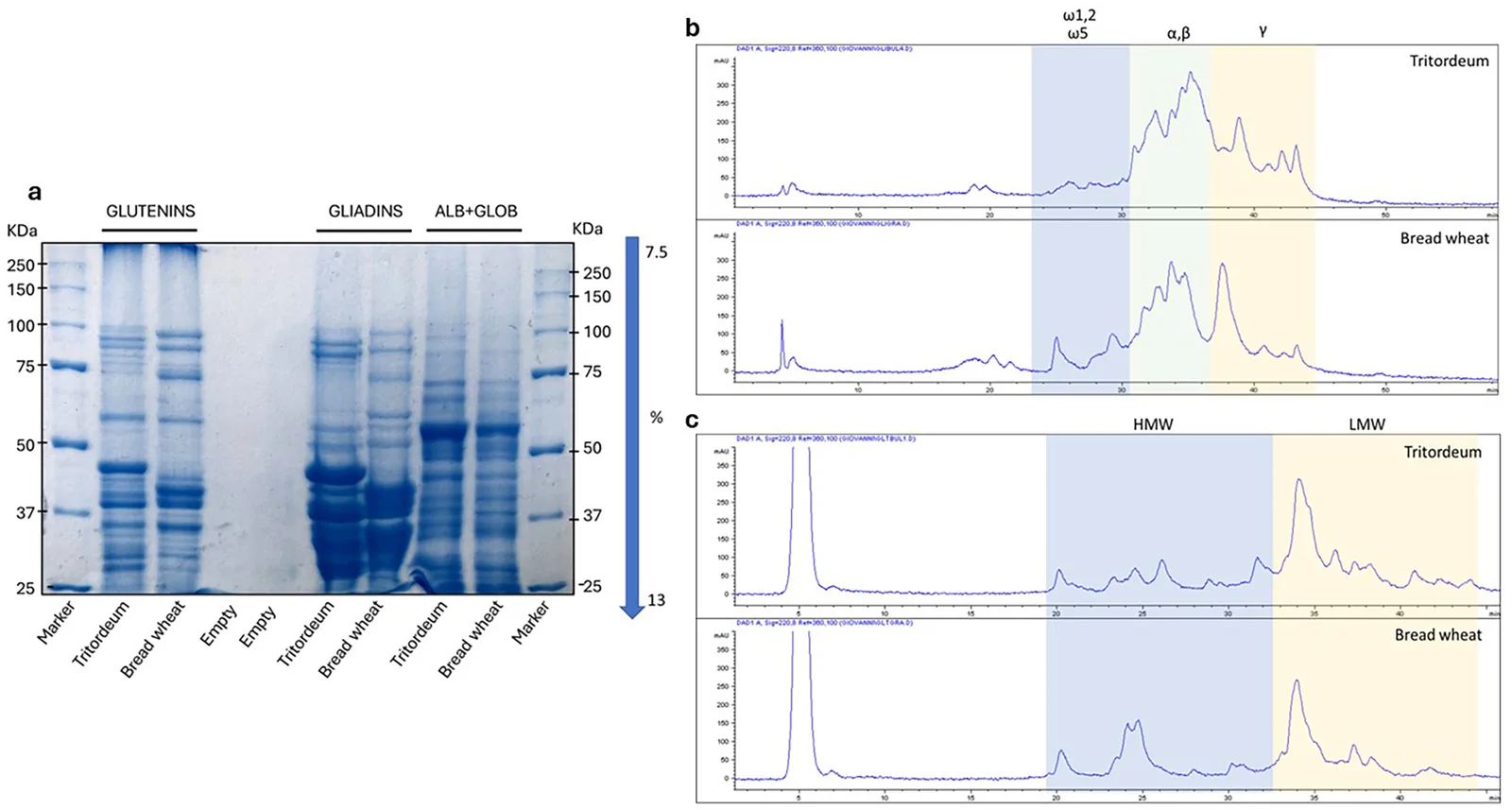
Protein characterization of the roller milled flours: (a) SDS-PAGE of the Osborne fractions; (b) RP-HPLC gliadin fraction; (c) RP-HPLC glutenin fraction. ALB, albumin; GLOB, globulin; HMW-GS, high-molecular-weight glutenin subunits; LMW-GS, low-molecular-weight glutenin subunits.

**Fig. 2:**
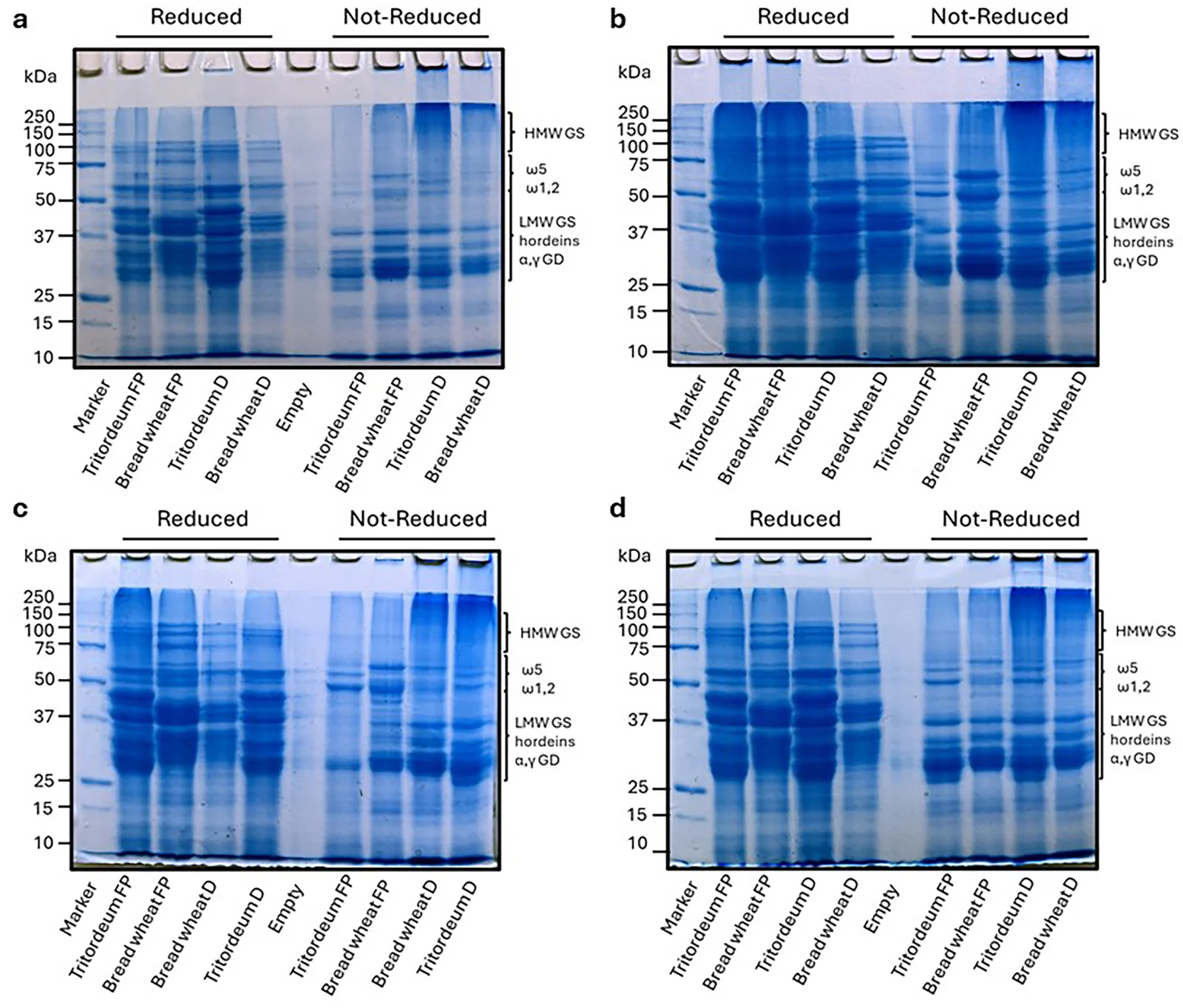
SDS-PAGE of the doughs (D) and final products (FP) of a) pasta; b) breadsticks; c) bread; d) pizza bases, produced with the roller-milled flours.

**Fig. 3:**
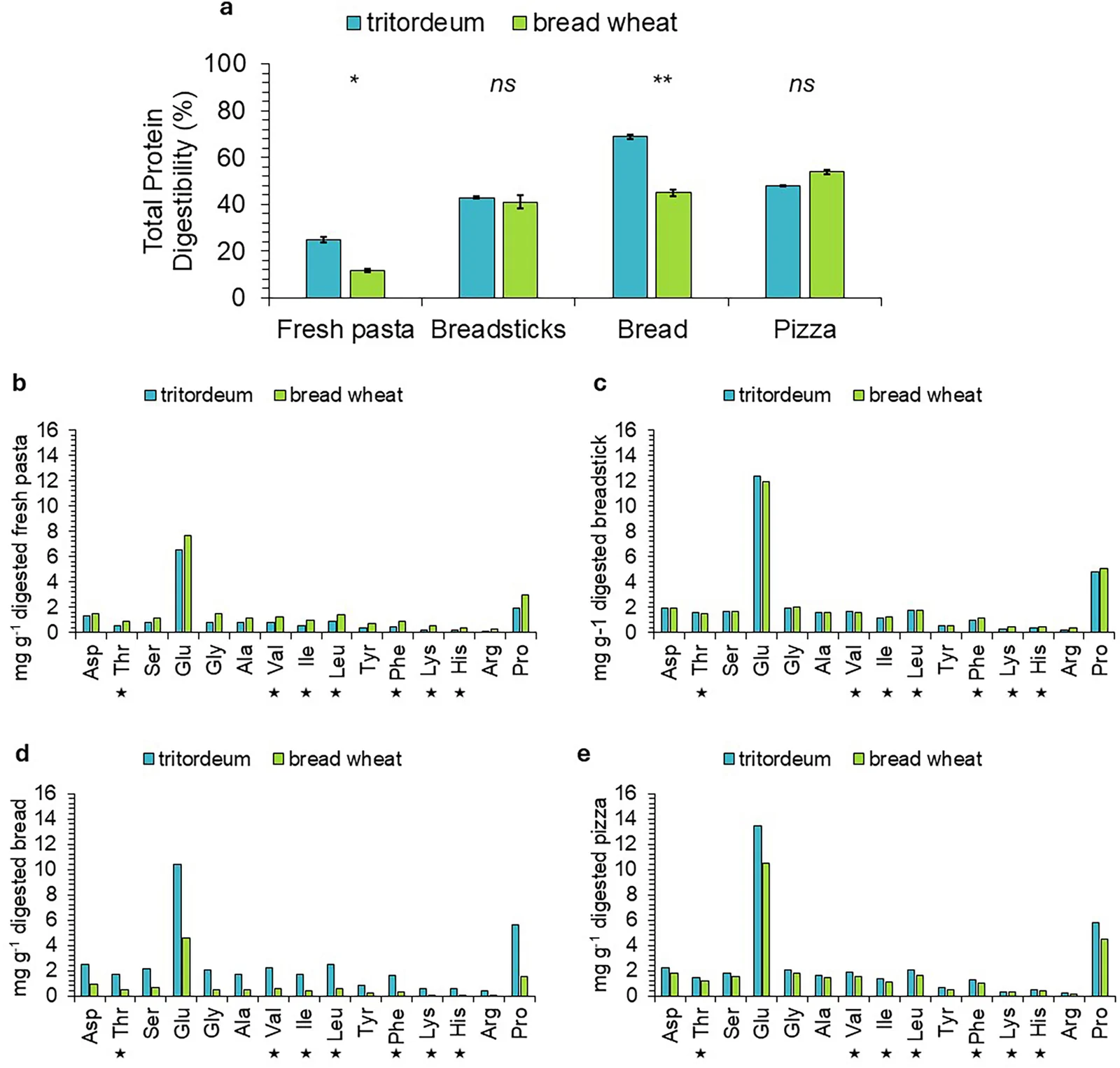
Protein digestibility of the roller-milled samples after simulated gastro-duodenal digestion based on a) total protein digestibility (%). Statistical significance: *** *p*-value ≤ 0.001; ** *p*-value ≤ 0.01; * *p*-value ≤ 0.05; ns, not significant (*p*-value > 0.05). Error bars represent the standard deviation (n = 2 biological replicates, each analyzed in duplicate); b) the individual amino acid digestibility of the fresh pasta; c) the individual amino acid digestibility of the breadsticks; d) the individual amino acid digestibility of the bread; e) the individual amino acid digestibility of the pizza bases. ★ Essential amino acid. IE-HPLC analyses were performed as single measurements on a pooled digest.

The RP-HPLC chromatograms of the gliadins (Fig. 1b) showed the characteristic separation of ω-, α/β-, and γ-gliadins. The overall profiles of the tritordeum and bread wheat varieties were qualitatively different, thus confirming the SDS-PAGE results. The obtained RP-HPLC chromatograms were similar to those of Landolfi et al. (2021), where the tritordeum exhibited a considerably lower number of ω-gliadins, which are known IgE-binders (Piboonpocanun et al., 2021).

An important parameter used to determine the quality of flour and its bread-making aptitude is the composition of its glutenin subfraction (HMW/LMW ratio). Fig. 1c shows the RP-HPLC chromatogram of the glutenin fraction. Again in this case, the two profiles looked different, especially with reference to the HMW-GS fraction, where the tritordeum variety showed more peaks than the variety of bread wheat, albeit with a lower intensity. Bread wheat exhibited a relatively higher proportion of HMW-GS than tritordeum, as confirmed by the significantly higher HMW/LMW-GS ratios observed in both milling systems (Table 1). These results are consistent with the SDS-PAGE patterns, where bands in the HMW region appeared less pronounced in the tritordeum variety.

Another factor that affects the technological properties of flour is the gliadin-to-glutenin ratio (GLI/GLU), which is widely recognized as a key factor in influencing the bread-making performance of wheat varieties. Glutenins and gliadins play distinct and complementary roles within the viscoelastic gluten network. Because of their ability to form large polymeric structures, glutenins establish a continuous protein network that provides dough with elasticity and strength. Gliadins instead act primarily as plasticizing components, thereby contributing to dough viscosity and extensibility (Wieser et al., 2023). Therefore, an appropriate balance between these protein fractions is essential to achieve the optimal combination of viscosity, elasticity, and mechanical strength required for the development of dough and its bread-making performance (Dhaka and Khatkar, 2015). The GLI/GLU ratios (Table 1) showed comparable values for the two varieties. The tritordeum variety displayed a slightly higher GLI/GLU ratio than that of bread wheat (1.67 vs 1.46) in the roller-milled samples, whereas the opposite trend was observed in the stone-milled flours (1.29 vs 2.32), although these differences were not statistically significant.

Differences in the gluten protein distribution were observed, despite the higher total protein content (Table 1) of the tritordeum flours (17.2–17.5% dw) than those of the bread wheat (12.7–13.7% dw). Similarly, the tritordeum variety showed a higher gluten content (33.2–39.6% dw) than bread wheat (29.2–30.0% dw). However, the lower HMW/LMW-GS and the comparable GLI/GLU ratios show that the higher protein content in the tritordeum variety was not associated with a proportional increase in polymer-forming glutenin subunits.

### 3.2 Rheological traits

The rheological properties of the flours were assessed, using complementary methods, to obtain information on the gluten aggregation behavior under high-shear conditions (GlutoPeak), dough development and stability during mixing (Farinograph), and on dough resistance to deformation and extensibility under biaxial extension (Alveograph) (Table 2).

**Table 2:** Gluten aggregation, mixing, and extensional and pasting properties of the tritordeum and bread wheat flours obtained from roller and stone milling.

Analysis	Parameter	Roller milling				Stone milling			
		Tritordeum		Bread wheat		Tritordeum		Bread wheat	
Gluten aggregation properties (from the GlutoPeak)	Maximum peak time (s)	76.5 ± 4.9	b	103.0 ± 1.4	a	53 ± 0.01	c	53.5 ± 3.5	c
	Maximum torque (GPU)	59.0 ± 0.01	b	47.5 ± 0.7	d	64.0 ± 1.4	a	53.0 ± 1.4	c
	Total energy (GPE)	2509 ± 51	a	2278 ± 42	a	3987 ± 2231	a	3659 ± 2184	a
Mixing properties (from the Farinograph)	Water absorption 500 FU (%)	60.3 ± 0.1	b	53.8 ± 0.2	d	66.6 ± 0.1	a	58.4 ± 0.01	c
	Development time (min)	3.55 ± 0.07	a	1.55 ± 0.07	b	3.85 ± 0.21	a	2.50 ± 0.99	ab
	Stability (min)	4.10 ± 0.01	b	4.50 ± 0.01	a	3.80 ± 0.01	c	4.50 ± 0.14	a
	Degree of softening 10 min (FU)	83.5 ± 2.1	a	77.0 ± 1.4	a	75.5 ± 10.6	a	61.0 ± 7.1	a
	Degree of softening 12 min (FU)	88.5 ± 0.7	ab	96.5 ± 0.7	a	85.0 ± 7.1	ab	81.0 ± 4.2	b
Extensional properties (from the Alveograph)	W (10E-4J)	181 ± 5.8	a	175 ± 3.6	a	174 ± 5.0	a	128 ± 5.0	b
	P/L	0.57 ± 0.01	c	0.60 ± 0.05	c	1.12 ± 0.04	a	0.99 ± 0.03	b
	Ie (%)	37.3 ± 0.5	b	53.8 ± 0.2	a	32.9 ± 0.7	c	38.5 ± 1.6	b
Pasting properties (from the Micro Visco-Amylo-Graph)	Pasting time (s)	952 ± 14	a	677 ± 48	b	968 ± 14	a	658 ± 3	b
	Pasting temperature (°C)	77.3 ± 0.7	a	65.2 ± 0.4	b	78.2 ± 0.8	a	65.0 ± 0.4	b
	Maximum viscosity time (s)	1314 ± 8	a	1257 ± 18	b	1293 ± 1	a	1253 ± 1	b
	Maximum viscosity (BU)	265 ± 5	b	293 ± 4	a	221 ± 0.01	c	295 ± 3	a
	Peak temperature (°C)	95.2 ± 0.3	a	92.6 ± 0.9	b	94.4 ± 0.1	a	92.4 ± 0.1	b
	Final viscosity (BU)	573 ± 15	a	596 ± 4	a	430 ± 4	b	581 ± 4	a
	Breakdown (BU)	48.0 ± 1.4	c	129.0 ± 1.4	a	51.0 ± 0.01	c	124.0 ± 1.4	b
	Setback (BU)	356 ± 11	b	432 ± 8	a	260 ± 4	c	410 ± 2	a

W, strength; P, tenacity; L, extensibility; Ie, elasticity index. Means followed by different letters in the same row are significantly different, according to the REGW-F test (*p*-value < 0.05).

As regards the gluten aggregation properties, the results showed that the effect of cereal type partly depended on the milling system. The tritordeum variety exhibited a significantly shorter maximum peak time and a higher maximum torque than that of bread wheat in the roller-milled samples, thus indicating faster protein aggregation and the formation of an aggregate with a higher maximum consistency. However, these differences were not accompanied by a significant variation of the total aggregation energy, which integrates the torque developed during the aggregation phase.

The maximum torque in wheat flour has been associated with the total protein and gliadin content, whereas the aggregation time and energy have been associated with glutenins and highly polymerized glutenin fractions (Marti et al., 2015). In the present study, the tritordeum variety showed a lower relative proportion of HMW-GS, whereas the GLI/GLU ratio did not differ significantly from that of the bread wheat variety (Table 1). Consequently, the higher maximum torque observed for the tritordeum variety should not be interpreted as evidence of a stronger gluten network; rather, it may reflect differences in matrix-water interactions, in part due to the high total protein and fiber content (Table 1). The response may also be influenced by differences in the presence of hordein-type proteins in tritordeum with different aggregation properties from those of wheat (Liu et al., 2025). Overall, these compositional features could promote the formation of a highly consistent aggregate, without necessarily resulting in a continuous, extensible, and mechanically stable dough network. In stone-milled samples, the maximum peak time was similar for the two cereal varieties, thus suggesting that whole-grain components may have modified the aggregation kinetics, particularly in the bread wheat, thereby eliminating the difference observed between the roller-milled samples. A similar behavior was observed by Malegori et al. (2018), who compared refined bread wheat and whole-meal flours. Instead, the maximum torque remained significantly higher in the tritordeum variety under both milling conditions, thus indicating that this index was the one, among the GlutoPeak parameters, that most consistently differentiated the two cereals.

In the dough system, the tritordeum variety exhibited a significantly higher water absorption than bread wheat under both milling conditions in the farinograph analysis (Table 2). The higher fiber and protein content (Table 1) may have reduced the amount of water readily available for gluten protein hydration and protein development. This mechanism may also partly explain the longer development time required by tritordeum to reach the target consistency of 500 FU. The tritordeum dough showed a significantly lower mixing stability than bread wheat dough, under both milling conditions, to reach the optimum water absorption, thus suggesting that, once formed, the tritordeum dough network would be less able to maintain its optimum consistency under continuous mechanical stress. The lower relative abundance of HMW-GS (Table 1) may have contributed to this behavior, as these subunits might promote intermolecular connectivity and the formation of large glutenin polymers involved in dough strength and mixing tolerance. Conversely, the comparable GLI/GLU ratios (Table 1) may indicate that the difference in mixing stability might not be associated with a substantial change in the overall balance of the gliadins or glutenins, but more likely with the qualitative composition and organization of the glutenin fraction. No significant differences between the two varieties were detected regarding the degree of softening after either 10 or 12 min within the same milling system. Therefore, although tritordeum required a longer time to develop, and it maintained its optimum consistency for a shorter period, its subsequent softening behavior was comparable with that of bread wheat under the considered experimental conditions.

Regarding the extensional properties, dough strength (W) and the balance between tenacity and extensibility (P/L) were affected by the milling system (Table 2). No significant differences in either W or P/L were observed between the two considered varieties in the roller-milled flours. Instead, tritordeum exhibited significantly higher W and P/L values than bread wheat in the stone-milled samples. However, tritordeum showed a significantly lower elasticity index under both milling conditions, thus indicating a lower ability of the dough to recover its original configuration after deformation.

When considered together, these results indicate that the stone-milled tritordeum dough was more resistant to deformation and more unbalanced toward tenacity, but not necessarily more elastic or characterized by a more cohesive gluten network than that of bread wheat. Therefore, the higher P/L value may primarily reflect reduced extensibility rather than intrinsically superior gluten strength. This interpretation is consistent with the protein profile (Table 1). The lower relative proportion of HMW-GS in the considered tritordeum variety may have limited its elastic recovery and intermolecular connectivity within the gluten network, whereas the absence of significant differences in the GLI/GLU ratio would suggest that its rheological behavior could not be explained by considering a simple shift in the overall monomeric-to-polymeric protein balance. Hordein-type proteins may have contributed to the distinctive response of tritordeum. Indeed, C-hordeins, which lack cysteine residues, do not directly take part in intermolecular disulfide-bond formation (Balakireva and Zamyatnin, 2016). Therefore, their presence may affect dough viscosity, without providing the covalent linkages required for the formation of an elastic glutenin network. The relatively high W and P/L values of the stone-milled tritordeum probably resulted from the combined effects of protein composition, fiber content, and water distribution rather than from a stronger gluten network per se.

The relatively high W values and the milling-dependent variation in P/L observed in the present study all fall within the broad technological variability reported for tritordeum. Overall, comparison with the literature has confirmed that tritordeum is characterized by a lower mixing stability and elastic gluten network than bread wheat, whereas its alveograph strength and tenacity-to-extensibility balance are highly genotype-dependent (Martín et al., 1999; Wiwart et al., 2025; Landolfi and Blandino, 2023).

As regards starch, the pasting properties of the tritordeum variety differed markedly from those of the bread wheat, irrespective of the milling system (Table 2). Tritordeum exhibited higher pasting temperatures and higher temperatures at maximum viscosity, thereby indicating that a higher temperature and a longer heating period were required before viscosity could develop to any great extent. In addition, the maximum viscosity was significantly lower in the tritordeum variety under both milling conditions. This profile suggests slower and more restricted swelling of the starch granules and a lower overall thickening capacity of the flour suspension. These findings are consistent with the distinctive pasting behavior recently reported for isolated tritordeum starch by Berski et al. (2024). In their study, tritordeum starch showed the highest pasting temperature, the longest time to reach peak viscosity, and the lowest peak viscosity of all the examined cereal starches. The authors attributed this behavior to the high resistance of tritordeum starch granules to swelling and to slow water diffusion into the granules, possibly associated with their relatively high lipid content and starch-lipid complexes. They also found that tritordeum starch was characterized by smaller granules than wheat starch, a feature that may further contribute to restricting swelling behavior. The studied tritordeum variety also exhibited significantly lower breakdown values than the bread wheat under both milling conditions (Table 2). This result is in close agreement with that of Berski et al. (2024), who observed no measurable breakdown for isolated tritordeum starch and described its hot paste as highly stable. However, in our case, the lower breakdown of the tritordeum variety might be interpreted as the consequence of delayed and restricted swelling, which limited any subsequent granule disruption during the high-temperature holding stage. Our setback values were also significantly lower in tritordeum than in bread wheat, thereby suggesting a reduced increase in viscosity during cooling and a lower short-term tendency of the starch-containing system to reassociate. This result is consistent with the slow retrogradation kinetics reported for isolated tritordeum starch by Berski et al. (2024). The same authors suggest that this was related to the high lipid content of tritordeum starch, which may restrict polymer mobility and interfere with starch-chain reassociation.

### 3.3 Technological traits

We evaluated the technological performance of the investigated tritordeum variety in four product models, namely fresh pasta, breadsticks, bread, and pizza bases, to determine whether the differences in the flour composition and rheological behavior translated into product-specific quality attributes (Table 3). Products characterized by markedly different structural requirements were selected to assess the suitability of the tritordeum variety for processing across a range of cereal-based applications.

**Table 3:** Quality characteristics of the products derived from the tritordeum and bread wheat flours obtained from roller and stone milling.

Product	Parameter	Roller milling				Stone milling			
		Tritordeum		Bread wheat		Tritordeum		Bread wheat	
Fresh pasta	Weight increase (%)	92.59 ± 2.74	a	75.42 ± 2.81	c	66.79 ± 1.76	b	90.56 ± 3.99	a
	Cooking loss (% dw)	6.40 ± 0.90	bc	5.29 ± 0.64	c	7.86 ± 1.26	ab	8.41 ± 0.63	a
	Firmness (N)	5.95 ± 0.86	a	5.76 ± 0.60	a	5.65 ± 0.74	a	4.55 ± 0.57	b
	Work of shear (g cm)	31.31 ± 3.87	a	28.49 ± 1.58	ab	31.90 ± 5.80	a	23.31 ± 3.15	b
	L*	79.60 ± 0.65	a	78.20 ± 0.77	b	67.08 ± 0.64	c	67.66 ± 1.08	c
	a*	2.57 ± 0.46	d	3.78 ± 0.08	c	9.15 ± 0.29	a	7.76 ± 0.20	b
	b*	31.84 ± 0.89	a	17.67 ± 0.31	d	30.36 ± 1.06	b	21.55 ± 0.27	c
Breadsticks	Hardness (N)	17.74 ± 2.42	a	13.19 ± 3.18	c	17.28 ± 1.94	a	14.98 ± 2.01	b
	Toughness (N mm^−1^)	19.23 ± 3.97	a	10.49 ± 2.57	b	17.32 ± 5.29	a	12.81 ± 2.88	b
	L*	65.13 ± 0.67	a	59.00 ± 1.06	b	53.00 ± 2.43	c	51.21 ± 0.77	d
	a*	11.10 ± 0.34	d	15.20 ± 0.73	c	15.92 ± 1.10	b	17.32 ± 0.32	a
	b*	38.43 ± 0.42	a	37.46 ± 0.33	a	32.43 ± 2.88	b	32.38 ± 0.65	b
Bread	Specific volume (mL g^−1^)	3.17 ± 0.14	b	3.92 ± 0.08	a	2.65 ± 0.07	c	2.63 ± 0.09	c
	Firmness (N)	13.08 ± 1.74	c	9.93 ± 1.35	d	15.49 ± 1.77	b	18.91 ± 1.60	a
	L* crust	42.97 ± 4.12	a	42.45 ± 4.19	a	36.71 ± 2.40	b	41.91 ± 3.81	ab
	a* crust	15.37 ± 0.87	a	15.54 ± 0.30	a	14.78 ± 0.85	a	15.36 ± 0.43	a
	b* crust	23.73 ± 3.50	a	23.10 ± 3.95	a	16.01 ± 2.80	b	22.12 ± 3.16	a
	L* crumb	66.06 ± 1.77	a	65.76 ± 2.47	a	58.15 ± 1.87	b	58.94 ± 1.41	b
	a* crumb	0.55 ± 0.22	b	1.41 ± 0.12	b	4.28 ± 1.70	a	3.81 ± 0.10	a
	b* crumb	21.16 ± 0.65	a	8.48 ± 0.67	d	20.12 ± 0.56	b	14.30 ± 0.39	c
Pizza bases	Firmness (N)	32.63 ± 10.02	b	64.57 ± 15.89	a	38.43 ± 10.45	b	61.27 ± 7.84	a
	L* up	60.03 ± 16.69	ab	64.15 ± 10.28	a	49.48 ± 12.35	b	60.46 ± 10.81	ab
	a* up	7.36 ± 3.12	b	9.34 ± 2.44	b	12.90 ± 2.30	a	9.79 ± 3.14	b
	b* up	23.66 ± 5.80	a	18.14 ± 3.87	b	22.53 ± 6.33	ab	18.10 ± 5.03	b
	L* bottom	72.75 ± 2.67	a	73.38 ± 6.39	a	58.86 ± 10.30	c	65.89 ± 5.53	b
	a* bottom	2.56 ± 1.52	c	5.29 ± 2.90	b	8.35 ± 3.24	a	7.20 ± 2.64	ab
	b* bottom	24.70 ± 2.14	a	15.39 ± 4.45	b	23.06 ± 2.70	a	17.29 ± 3.06	b

L* = luminosity; a* = redness; b* = yellowness. Means followed by different letters in the same row are significantly different, according to the REGW-F test (*p*-value < 0.05).

Tritordeum pasta exhibited a significantly higher weight increase than bread wheat in roller-milled samples, thus reflecting a greater water uptake, whereas the opposite trend was observed after stone milling. The smaller weight increase of stone-milled tritordeum may be related to its higher fiber content and to the resulting changes in its matrix organization: bran-rich particles may compete with starch and proteins for water, reduce the relative amount of swellable starch and hinder water diffusion within the pasta structure. The cooking loss did not differ significantly between the two varieties within either milling system, although the stone-milled samples showed higher values than their roller-milled counterparts. This trend may reflect the greater presence of bran particles, which can disrupt matrix continuity and facilitate the release of soluble material during cooking (Manthey and Schorno, 2002). Overall, the two varieties showed a comparable ability to retain solids during cooking. The relatively short cooking time may also have limited the disintegration of the matrix and reduced the magnitude of the differences in the cooking losses. Firmness and work of shear did not differ significantly between the roller-milled tritordeum and bread wheat pasta. However, the stone-milled tritordeum pasta was significantly firmer and required more work of shear than the corresponding bread wheat samples. The relatively firm and mechanically resistant structure of tritordeum pasta, particularly after stone milling, may reflect the combined effects of its higher protein and fiber contents (Table 1), restricted starch swelling (Table 2), and lower water uptake during cooking. The greater firmness and work of shear should not be interpreted as evidence of superior gluten quality; indeed, the rheological results reported in Table 2 indicate lower elasticity and mixing stability for tritordeum. Therefore, the pasta texture was likely governed more by the organization of the overall protein-starch-fiber matrix than by the formation of a stronger gluten network. To the best of the authors’ knowledge, this is the first study to have investigated the technological performance of pasta produced exclusively from tritordeum flour.

As regards baked goods, the effect of tritordeum on the product texture depended significantly on the structural requirements of the baked product. Breadsticks prepared with the tritordeum variety were consistently harder and tougher than those made with the bread wheat variety, irrespective of the milling system, thus indicating the formation of a denser and more mechanically resistant structure. The lower level of extensibility and elasticity of tritordeum dough do not represent a technological limitation for low-moisture products, since breadsticks do not require extensive gas-cell expansion or stabilization. A compact matrix may instead contribute toward achieving a firm and fracture-resistant texture, which is typical of this product category (Guiné, 2022). The higher fiber content and water absorption of tritordeum may also have modified the water distribution during baking, as reported for bran-enriched cereal products (Curti et al., 2013). Its restricted starch swelling may have contributed to the final structure, although this mechanism remains to be confirmed in breadsticks. As direct comparisons between tritordeum and bread wheat breadsticks remain scarce, these results provide further evidence of the suitability of the studied tritordeum variety for low-moisture baked products.

A different response of the milling system was observed in the bread, where the ability of the dough to expand and retain gas was central to product quality. The tritordeum bread had an approximately 20% lower specific volume than the bread wheat bread in the roller-milled samples. This volume agrees with previous reports of reduced loaf volumes of tritordeum, although considerable genotypic variability has been documented (Martín et al., 1999; Wiwart et al., 2025; Landolfi and Blandino, 2023). The lower loaf expansion of roller-milled tritordeum was consistent with its lower dough elasticity and mixing stability (Table 2) and with the protein profile reported in Table 1. Although the tritordeum variety had a higher total protein content, it showed a lower relative abundance of HMW-GS subunits (Table 1). Since HMW-GS contribute to gluten strength, their smaller proportion may have limited the formation of a cohesive and extensible network capable of retaining gas during proofing and baking. The comparable GLI/GLU ratios between the two cereal varieties also suggest that the difference in bread-making performance was not be mainly related to the overall balance between gliadins and glutenins, but rather to the qualitative composition of the glutenin fraction, and the HMW/LMW-GS balance in particular. Nevertheless, this interpretation should be considered together with the effects of fiber content, hydration levels, water distribution, and milling conditions, which also influence dough development and loaf volume. The roller-milled tritordeum bread was also firmer than the bread wheat sample, in line with its lower specific volume. A similar tendency was reported by Wiwart et al. (2025), who found a higher average crumb hardness in tritordeum bread than control.

Stone milling reduced the specific volume of both varieties and eliminated the difference between them. The incorporation of bran- and germ-rich fractions likely disrupted gluten continuity, increased competition for water, and restricted gas-cell expansion (Curti et al., 2013), particularly in the bread wheat variety. Despite their similar specific volumes, the stone-milled tritordeum bread was less firm than the bread wheat samples, thereby showing that crumb firmness was not determined by the loaf volume alone. Differences in the fiber characteristics, water distribution, and starch behavior might also have contributed; the higher water absorption of the tritordeum variety may have altered the water partitioning within the crumbs (Curti et al., 2013), while the lower setback suggests a lower tendency toward starch reassociation during cooling, which may have limited firming of the crumbs.

Finally, the pizza bases prepared with the tritordeum variety were softer than those made with bread wheat under both milling conditions. Such differences may reflect the combined effects of flour composition, hydration, protein-network organization, and starch transformations during baking. Indeed, other studies have shown that dough evolution during leavening and baking and the composition of milling streams both influence the structural and textural properties of pizza bases (Covino et al., 2023; Mahajan et al., 2025).

Overall, color was affected by the cereal variety, milling system, and product surface. The tritordeum flour (Table 2) had significantly higher b* values than both the roller- and stone-milled bread wheat flours (+56 and + 30%). This is consistent with the higher carotenoid content reported for tritordeum, particularly its greater lutein concentration (Sardella et al., 2026). The L* and a* values of the flours were mainly affected by the milling system. Tritordeum showed a stronger yellow component in the breadcrumbs and on the lower surface of the pizza bases under both milling conditions, and on the upper surface of the roller-milled pizza bases (Table 3). However, the two varieties did not differ in b* in the breadsticks, while the tritordeum products were lighter and less red. Stone milling reduced product lightness, thereby reflecting the greater contribution of peripheral grain tissues, and partially masked the yellow pigmentation associated with tritordeum carotenoids in some products. Moreover, the tritordeum pasta showed higher b* values than the bread wheat pasta under both milling conditions. Redness depended on the milling process: the roller-milled tritordeum pasta showed a lower a* value than the bread wheat pasta, whereas the opposite trend was observed in the stone-milled samples.

### 3.4 Protein profiling of the doughs, fresh pasta, and baked products

As no statistically significant differences were observed in the protein profiles of the roller- and stone-milled flours (Table 1), the subsequent protein profiling and protein and starch digestibility analyses were only carried out on the roller-milled dough and derived product samples. The SDS-PAGE analysis of the proteins extracted from both the doughs and final baked products under reducing conditions showed more extractability and profile complexity than those extracted under non-reducing conditions (Fig. 2). This is because the glutenin protein family, which is rich in cysteines, forms inter and intra disulfide bridges between each other and with gliadins (Wieser et al., 2023). Protein complexes with a molecular weight > 250 kDa are difficult to extract under non-reducing conditions and exhibit poor migration through polyacrylamide gel, and this results in a suboptimal and incomplete electrophoretic profile (Fig. 2). Although no macroscopic differences were observed over the various technological processes, it was still possible to discriminate between the products derived from the two varieties.

### 3.5 In vitro static simulated digestion of the fresh pasta and baked products and determination of the protein hydrolysis degree and amino acid release

After the structural characterization, the roller-milled products were subjected to a standardized static *in vitro* gastro-duodenal digestion model which simulated the digestive conditions of a healthy adult. Overall, the extent of protein hydrolysis, expressed as total protein digestibility after simulated gastro-duodenal digestion varied, depending on both the flour variety and the type of product (Fig. 3). Bread and pizza exhibited the highest total protein digestibility values for both roller-milled flours (Fig. 3a), thus suggesting that the structural characteristics of these products may facilitate enzymatic access to protein substrates. The tritordeum-based bread and pasta formulations exhibited significantly higher protein digestibility than their bread wheat counterparts. In contrast, differences between the two varieties were less pronounced and statistically not significant in the breadsticks, while bread wheat displayed slightly higher digestibility in the pizza samples, although no statistically significant difference was observed between the two flours.

Further insights were obtained by analyzing the amino acid release in the soluble duodenal fraction. Glutamine and proline were the most abundant amino acids among those detected. This result is consistent with the amino acid composition of gluten proteins, particularly gliadins and glutenins, which are typically rich in these residues. The tritordeum formulations in the bread and pizza samples (Fig. 3b and c) exhibited a higher apparent release of essential amino acids than the corresponding bread wheat formulations. This descriptive observation may be related to differences in protein network organization between the two varieties, which could affect not only the overall extent of protein hydrolysis but also the profile of amino acids released during digestion. Although the variety of tritordeum developed a less stable and elastic gluten network than that of bread wheat, its less interconnected protein structure may have increased the accessibility of digestive enzymes to storage proteins, potentially contributing to the higher protein digestibility observed in the tritordeum-based formulations. However, given that the amino acid analyses were performed as single measurements on a pooled digest, these differences should be considered as descriptive observations rather than statistically established effects. Collectively, these findings indicate that the structural features that govern dough rheology cannot be used to predict protein digestibility in the final food matrix and highlight protein network organization as a key determinant of both dough functionality and nutrient bioaccessibility in cereal-based foods.

### 3.6 Starch digestion monitoring in bread

Starch digestibility varies significantly in foods, and it is governed by several factors, including the physical structure of the food matrix and interactions between its chemical components (Smith et al., 2015).

The kinetics of free glucose release from the roller-milled tritordeum and wheat bread samples were comparable (Fig. 4a), indicating similar *in vitro* starch hydrolysis profiles under the experimental conditions applied. However, the RHS fraction was significantly lower in the tritordeum bread samples. Together, the FGS and RHS fractions corresponded to approximately 51 and 58 g starch equivalents 100 g^−1^ bread for tritordeum and bread wheat, respectively, after 300 min of duodenal digestion (Fig. 4b). This evidence suggests a lower glycemic load after consuming tritordeum-based bread. The RS content of bread wheat (0.7 ± 0.2 g 100 g^−1^) was consistent with values reported for conventional wheat bread (0.8 g 100 g^−1^), whereas tritordeum bread exhibited a markedly higher RS content (3.5 ± 0.6 g 100 g^−1^), thus suggesting a greater fraction of starch resistant to enzymatic hydrolysis (Amaral et al., 2022). This finding is consistent with the different starch functionalities observed for the studied tritordeum variety, which may promote the formation of starch structures that are less susceptible to enzymatic digestion during bread-making. These differences in *in vitro* starch digestibility may have implications for postprandial glycemic responses; however, such effects cannot be established from the present static digestion model and would require confirmation *in vivo*. According to a previously published study (Smith et al., 2015), the digestion of starch and proteins influences each other. This effect is likely due to the formation of a gluten-starch complex during both the mixing and cooking processes. The breakdown of starch granules during digestion may increase protein digestibility, and the breakdown of the gluten network may positively impact starch digestion (Smith et al., 2015). Although static *in vitro* digestion protocols (e.g., INFOGEST) are widely used, they may underestimate starch hydrolysis. Unlike *in vivo* conditions, where salivary α-amylase remains active until the gastric pH drops below 4.5, static methods typically involve an immediate transition to pH 3. This rapid acidification prematurely inactivates the enzyme, and these methods fail to account for the prolonged starch digestion that occurs in the stomach, a process that is further modulated by the subject's physiology and the complexity of the food matrix. Nevertheless, the INFOGEST protocol can be considered a robust tool for comparative analysis within the framework of this research. Although it may lead to a potential underestimation of the absolute starch hydrolysis levels, its utility for benchmarking different formulations is well known. Indeed, static digestion models have been shown to yield consistent outcomes for more complex dynamic experiments when used for comparative purposes (Sagnelli et al., 2018). These findings indicate that the structural organization of cereal proteins influences not only the functionality of dough but also the nutritional fate of digestion products.

**Fig. 4:**
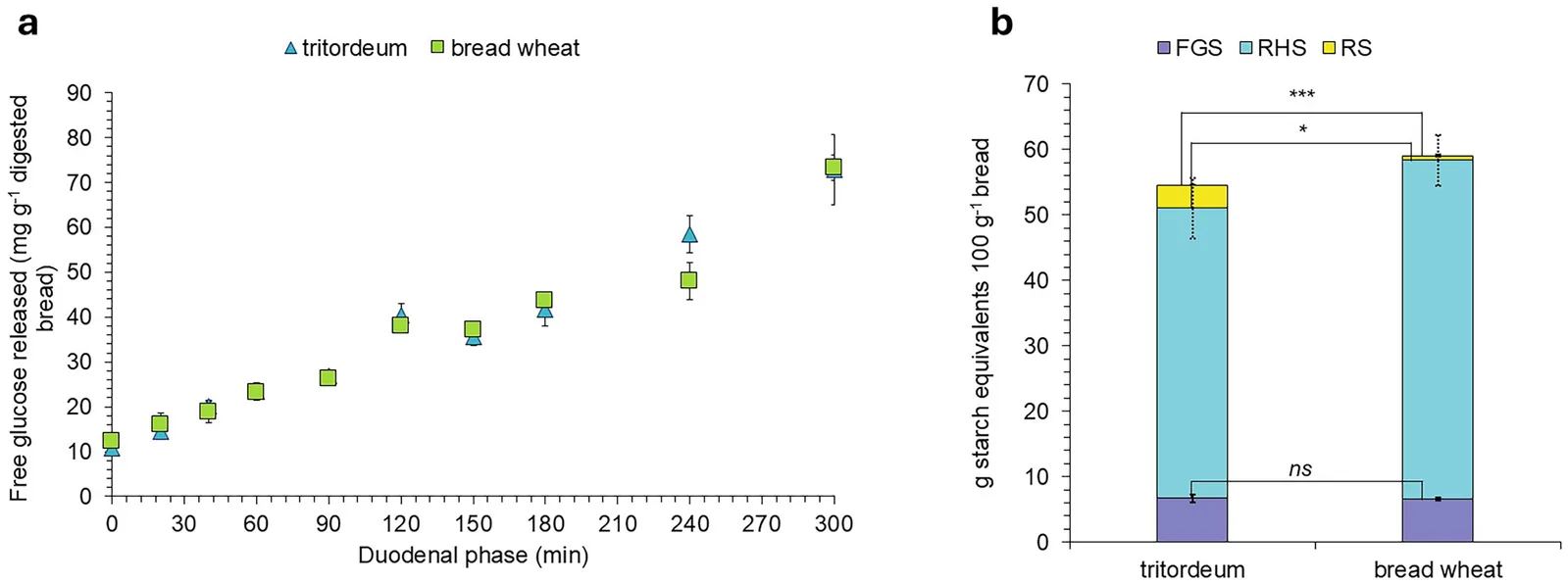
Starch digestibility of the bread produced from roller milling: a) free glucose released during 300 min of simulated duodenal digestion; b) starch fractions determined after 300 min of simulated duodenal digestion. FGS: free glucose-derived starch; RHS: residual hydrolysable starch; RS: resistant starch. Statistical significance: *** *p-*value ≤ 0.001; ** *p-*value ≤ 0.01; * *p-*value ≤ 0.05; ns, not significant (*p-*value > 0.05). Error bars represent the standard deviation (n = 2 biological replicates, each analyzed in duplicate).

## 4 Conclusions and limitations of the study

Overall, under the conditions investigated, the selected tritordeum material showed a distinct technological behavior from that of bread wheat, due to the combined effects of protein composition, fiber content, starch functionality, and milling conditions. Despite the higher total protein content and higher GlutoPeak maximum torque of the investigated tritordeum variety, it developed a less elastic and less stable dough network, consistent with its lower relative abundance of HMW glutenin subunits. These rheological characteristics limited gas retention and loaf expansion in the roller-milled bread, but they did not result in uniformly poorer product quality. The more compact structure of tritordeum in the low-moisture products, such as breadsticks, led to greater hardness and toughness, while it provided satisfactory cooking stability in fresh pasta and, in particular after stone milling, a firmer cooked texture. The considered tritordeum variety produced a softer structure in the pizza bases than bread wheat, thus showing that its weaker dough elasticity can be compatible with products in which high loaf expansion is not the main quality requirement.

The milling system further modulated these responses by altering the fiber content, and likely also the water distribution and matrix organization. Stone milling reduced the bread volume in both varieties, modified pasta hydration, and changed the relative texture of the bread and pizza bases.

Furthermore, tritordeum bread and fresh pasta produced from the roller-milled flour showed significantly higher protein digestibility than the corresponding bread wheat formulations, together with an apparent higher release of essential amino acids during simulated intestinal digestion. Tritordeum bread produced from roller-milled flour also exhibited substantially higher resistant starch content and a lower proportion of digestible starch, highlighting differences in starch digestibility between the two breads. Therefore, the technological performance of tritordeum cannot be predicted from just the flour composition or dough rheology, and it needs to be evaluated in relation to the structural requirements of the final product.

Overall, the selected tritordeum variety appears to be a versatile raw material, whose suitability depends on the specific product, and it should be assessed by considering both its technological performance and nutritional functionality, instead of assuming it is universally superior or inferior to bread wheat. However, the observed differences reflect the combined effects of flour characteristics and the formulation (hydration levels) and processing conditions adopted, and cannot be attributed exclusively to cereal identity. Since only one cultivar of each cereal was evaluated, further studies including multiple genotypes are needed to determine the extent to which the present findings can be generalized. Future research should also include a more comprehensive characterization of starch structural properties, including thermal behavior and starch granule morphology, to further elucidate the mechanisms underlying the observed differences in starch digestion, as well as sensory evaluation and consumer acceptance of tritordeum-based products.

## CRediT authorship contribution statement

Giovanni D’AURIA: Conceptualization, Formal analysis, Investigation, Methodology, Visualization, Validation, Writing – original draft.

Claudia SARDELLA: Conceptualization, Formal analysis, Investigation, Methodology, Visualization, Validation, Writing – original draft.

Andrea BRESCIANI: Formal analysis, Investigation, Methodology, Writing – original draft.

Felicia GRAZIUSO: Investigation, Methodology, Visualization, Writing – review and editing.

Chiara NITRIDE. Conceptualization, Supervision, Validation, Writing – review and editing.

Luisa PELLEGRINO: Methodology, Writing – review and editing.

Pasquale FERRANTI: Conceptualization, Resources, Supervision, Writing – review and editing.

Alessandra MARTI: Methodology, Resources, Supervision, Writing – review and editing.

Massimo BLANDINO: Conceptualization, Funding acquisition, Project administration, Resources, Methodology, Supervision, Visualization, Writing – review and editing.

## Data availability

Data will be made available on request.

## Funding sources

The work was supported by the Regione Piemonte (POR FESR 2014-2020), as a part of the DEUM project, and by the Italian Ministry of University and Research (National Recovery and Resilience Plan, Mission 4 Component 2 Investment 1.3, CUP D93C22000890001, funded by the European Union-NextGenerationEU) as a part of the ON Foods project.

## Declaration of competing interest

The authors declare that they have no known competing financial interests or personal relationships that could have appeared to influence the work reported in this paper.

## Abbreviations

a*, redness; AMG, amyloglucosidase; ANOVA, analysis of variance; b*, yellowness; dw, dry weight; FGS, free glucose-derived starch; GLI, gliadins; GLU, glutenins; HMW-GS, high-molecular-weight glutenin subunits; HPLC, high-performance liquid chromatography; IE, Ion-Exchange; Ie, elasticity index; L*, luminosity; LMW-GS, low-molecular-weight glutenin subunits; P/L, tenacity/extensibility ratio; REGW-F test, Ryan-Einot-Gabriel-Welsch F test; RH, relative humidity; RHS, residual hydrolysable starch; RID, reflectance index detection; RP, reversed-phase; RS: resistant starch; SDS-PAGE, sodium dodecyl sulfate-polyacrylamide gel electrophoresis; TAME, ρ-toluene-sulfonyl-L-arginine methyl ester; TCA, trichloroacetic acid; W, alveographic strength.

## Appendix A Supplementary data

Supporting information available for publication: Table S1, Particle size distribution of the flours used in the study; Figure S1. Visual comparison of the grains (A), the flours (B), and the products (C) evaluated in the study; Figure S2, Diagram of the experimental workflow and technological steps. The collected and analyzed raw materials (refined and semi-whole flours), doughs, and the final products (fresh pasta, breadsticks, bread, and pizza bases) are reported in the ovals; Figure S3, Gluten aggregation profiles of the refined tritordeum (gray dotted line) and bread wheat (gray line) flours, and of the semi-whole tritordeum (dotted black line) and bread wheat (black line) flours obtained from the GlutoPeak test; Figure S4, Pasting profiles of the refined tritordeum (gray dotted line) and bread wheat (gray line) flours, and of the semi-whole tritordeum (dotted black line) and bread wheat (black line) flours obtained from the Micro Visco-Amylo-Graph test.
