# Supplementary material for: From milling to digestion: matrix-driven effects of tritordeum and wheat flours on the technological quality and digestibility of cereal-based foods
Source: Curr Res Food Sci. 2026 Oct 12;13:101595. doi: 10.1016/j.crfs.2026.101595 (PMC13693066; doi:10.1016/j.crfs.2026.101595)
Supplement: Supplementary file 1 [file mmc1.docx]

**Table S1.**

Particle size distribution of the flours used in the study.

| **Type of flour** | **Milling method** | **200-300 µm** | **160-200 µm** | **112-160 µm** | **< 112 µm** |
| --- | --- | --- | --- | --- | --- |
|  |  |  |  |  |  |
| Refined flour | Roller milling | 0.4% | 8.7% | 26.8% | 64.1% |
| Semi-whole flour | Stone milling | 3.4% | 14.4% | 19.0% | 63.2% |

**Figure S1.** Visual comparison of the grains (A), the flours (B), and the products (C) evaluated in the study.

**Figure** **S2.** Diagram of the experimental workflow and technological steps. The collected and analyzed raw materials (refined and semi-whole flours), doughs and final products (fresh pasta, breadsticks, bread, and pizza bases) are reported in the ovals.

**Figure S3.** Gluten aggregation profiles of the refined tritordeum (gray dotted line) and bread wheat (gray line) flours, and of the semi-whole tritordeum (dotted black line) and bread wheat (black line) flours obtained from the GlutoPeak test.

Torque (BU)

| Time (s) | Tritordeum, semi-whole flour | Tritordeum pietra Run 2 | Tritordeum, refined flour | Tritordeum pietra Run 2 | Bread wheat, semi-whole flour | Tritordeum pietra Run 2 | Bread wheat, refined flour | Tritordeum pietra Run 2 |
| --- | --- | --- | --- | --- | --- | --- | --- | --- |
| 0 | 0 | 0 | 0 | 0 | 0 | 0 | 0 | 0 |
| 1 | 0 | 0 | 0 | 0 | 0 | 0 | 0 | 0 |
| 2 | 0.9 | 1.1 | 1.2 | 1.1 | 1.1 | 0.8 | 0.7 | 0.9 |
| 3 | 1.7 | 2.2 | 1.8 | 1.8 | 2 | 1.9 | 1.4 | 1.5 |
| 4 | 3.7 | 4.6 | 4.1 | 4 | 3.8 | 4 | 2.5 | 2.6 |
| 5 | 7.3 | 8.4 | 7.3 | 6.6 | 7.2 | 6.6 | 4.5 | 4.1 |
| 6 | 10.6 | 11.5 | 9.4 | 8.7 | 10.7 | 8.6 | 6.4 | 5.8 |
| 7 | 12.3 | 13 | 9.7 | 9.5 | 12.8 | 10.4 | 7.6 | 7 |
| 8 | 13.9 | 14.4 | 11 | 10.6 | 14.5 | 12.2 | 8.7 | 8.6 |
| 9 | 15.9 | 16.5 | 12.6 | 12.1 | 16.7 | 14.2 | 10.2 | 10.2 |
| 10 | 17.9 | 18.6 | 13.9 | 13.8 | 18.8 | 16.1 | 11.5 | 11.5 |
| 11 | 19.9 | 20.5 | 14.9 | 15.4 | 20.3 | 17.7 | 12.6 | 12.5 |
| 12 | 21.6 | 22 | 15.7 | 16.7 | 21.4 | 19.1 | 13.4 | 13.3 |
| 13 | 22.9 | 23 | 16.4 | 17.7 | 22.1 | 20.1 | 14 | 13.9 |
| 14 | 23.8 | 23.7 | 16.9 | 18.4 | 22.6 | 20.8 | 14.3 | 14.2 |
| 15 | 24.3 | 24.2 | 17.2 | 18.7 | 22.8 | 21.1 | 14.5 | 14.3 |
| 16 | 24.4 | 24.5 | 17.2 | 18.5 | 22.7 | 21.2 | 14.5 | 14.2 |
| 17 | 24.3 | 24.6 | 17.2 | 18.3 | 22.6 | 21.2 | 14.4 | 14.1 |
| 18 | 24.3 | 24.5 | 17.1 | 18.1 | 22.5 | 21.2 | 14.2 | 14 |
| 19 | 24.3 | 24.5 | 17.1 | 18 | 22.4 | 21.1 | 14.1 | 13.9 |
| 20 | 24.3 | 24.5 | 17.1 | 17.8 | 22.3 | 21.2 | 14 | 13.9 |
| 21 | 24.3 | 24.7 | 17.1 | 17.7 | 22.4 | 21.4 | 13.9 | 13.8 |
| 22 | 24.4 | 24.8 | 17 | 17.7 | 22.5 | 21.5 | 13.8 | 13.7 |
| 23 | 24.4 | 24.8 | 17 | 17.6 | 22.6 | 21.5 | 13.7 | 13.6 |
| 24 | 24.9 | 25 | 17 | 17.5 | 22.7 | 21.6 | 13.6 | 13.5 |
| 25 | 25.1 | 25.1 | 17 | 17.4 | 22.8 | 21.7 | 13.4 | 13.5 |
| 26 | 25.2 | 25.1 | 17 | 17.3 | 23 | 21.7 | 13.4 | 13.4 |
| 27 | 25.3 | 24.9 | 17 | 17.2 | 23.4 | 21.8 | 13.4 | 13.4 |
| 28 | 25.8 | 25.1 | 17 | 17.2 | 24.1 | 21.9 | 13.3 | 13.3 |
| 29 | 26.2 | 25.3 | 17 | 17.1 | 25.2 | 22.2 | 13.3 | 13.3 |
| 30 | 26.8 | 25.7 | 17 | 17.1 | 26.5 | 22.7 | 13.3 | 13.3 |
| 31 | 27.2 | 26.1 | 17.1 | 17 | 27.6 | 22.7 | 13.3 | 13.3 |
| 32 | 27.6 | 26.7 | 17.1 | 17 | 29 | 23.1 | 13.2 | 13.2 |
| 33 | 28.3 | 27.8 | 17.1 | 17 | 30.2 | 23.9 | 13.2 | 13.2 |
| 34 | 29.3 | 29.3 | 17.3 | 17 | 30.7 | 24.4 | 13.2 | 13.2 |
| 35 | 30.7 | 30.9 | 17.4 | 17 | 31.2 | 24.9 | 13.2 | 13.2 |
| 36 | 32.1 | 32.3 | 17.3 | 17 | 31.5 | 25.4 | 13.1 | 13.2 |
| 37 | 33.5 | 33.6 | 17.2 | 17 | 32.4 | 26.2 | 13.1 | 13.2 |
| 38 | 34.6 | 35.1 | 17.3 | 17.1 | 33.3 | 28 | 13.1 | 13.3 |
| 39 | 35.9 | 36.5 | 17.4 | 17.1 | 34.3 | 30.6 | 13.2 | 13.4 |
| 40 | 37.1 | 37.7 | 17.5 | 17.1 | 35.3 | 33.3 | 13.2 | 13.4 |
| 41 | 38.1 | 39 | 17.7 | 17.1 | 36.3 | 35.1 | 13.2 | 13.4 |
| 42 | 39.2 | 40.2 | 17.8 | 17.1 | 37.9 | 36.4 | 13.2 | 13.4 |
| 43 | 40.4 | 41.3 | 18.3 | 17.3 | 40 | 37.5 | 13.2 | 13.3 |
| 44 | 41.9 | 42.9 | 18.7 | 17.4 | 42.1 | 39.1 | 13.2 | 13.2 |
| 45 | 43.6 | 44.7 | 18.9 | 17.5 | 44.4 | 40.4 | 13.2 | 13.2 |
| 46 | 45.3 | 46.1 | 19 | 17.7 | 46.5 | 41.6 | 13.2 | 13.2 |
| 47 | 47.3 | 47.6 | 19.3 | 17.8 | 48.6 | 43.4 | 13.2 | 13.2 |
| 48 | 49.7 | 50.3 | 19.7 | 17.8 | 50.3 | 45.3 | 13.2 | 13.3 |
| 49 | 53.2 | 52.7 | 19.9 | 17.7 | 52.2 | 46.1 | 13.3 | 13.3 |
| 50 | 56.3 | 55.4 | 20.3 | 17.8 | 53.9 | 47 | 13.3 | 13.3 |
| 51 | 59.8 | 58.6 | 20.7 | 18 | 54.4 | 48.1 | 13.4 | 13.3 |
| 52 | 62.3 | 62.5 | 21.1 | 18.2 | 53.9 | 49.5 | 13.5 | 13.4 |
| 53 | 63.3 | 65 | 21.6 | 18.4 | 52.9 | 50.7 | 13.5 | 13.4 |
| 54 | 63.3 | 64.3 | 22.1 | 18.8 | 51.7 | 51.2 | 13.6 | 13.5 |
| 55 | 62.1 | 62.4 | 22.9 | 19.1 | 50.6 | 51.7 | 13.6 | 13.5 |
| 56 | 60.9 | 59.4 | 23.9 | 19.4 | 49.5 | 51.7 | 13.6 | 13.6 |
| 57 | 60.2 | 57.1 | 25.2 | 19.7 | 48.6 | 51.3 | 13.7 | 13.6 |
| 58 | 58.7 | 56.2 | 26.5 | 20 | 47.8 | 50.6 | 13.8 | 13.7 |
| 59 | 57.5 | 55.9 | 28.6 | 20.3 | 47 | 49.7 | 13.8 | 13.7 |
| 60 | 55.7 | 55.3 | 31.1 | 20.9 | 45.9 | 49 | 13.9 | 13.8 |
| 61 | 54.3 | 54.2 | 33.9 | 22 | 44.6 | 48.3 | 14 | 13.8 |
| 62 | 53.1 | 53.2 | 37.3 | 23 | 43.1 | 47.5 | 14 | 13.9 |
| 63 | 52 | 52.6 | 41 | 23.7 | 42.1 | 46.7 | 14.1 | 14 |
| 64 | 51.3 | 52.2 | 44.6 | 24.6 | 41.4 | 45.7 | 14.2 | 14 |
| 65 | 50.6 | 51.5 | 48.3 | 25.5 | 40.9 | 44.4 | 14.3 | 14.1 |
| 66 | 50 | 50.8 | 51.9 | 26.3 | 40.5 | 43.6 | 14.3 | 14.2 |
| 67 | 49.4 | 50.1 | 55.4 | 27.5 | 40.4 | 43 | 14.4 | 14.3 |
| 68 | 48.9 | 49.4 | 56.9 | 28.9 | 40.1 | 41.7 | 14.4 | 14.4 |
| 69 | 48.2 | 48.8 | 57.4 | 30.8 | 40 | 41 | 14.6 | 14.6 |
| 70 | 47.7 | 48.1 | 57.8 | 33.4 | 40 | 40.6 | 14.6 | 14.7 |
| 71 | 47.1 | 47.6 | 58.1 | 36.7 | 40 | 40.5 | 14.7 | 14.8 |
| 72 | 46.6 | 47 | 58.3 | 40.2 | 40 | 40.3 | 14.9 | 14.9 |
| 73 | 46.2 | 46.5 | 58.7 | 44.6 | 40 | 40.3 | 15 | 14.9 |
| 74 | 45.8 | 46 | 57.8 | 49 | 39.8 | 40.3 | 15.2 | 15.1 |
| 75 | 45.4 | 45.7 | 57.3 | 53.3 | 39.6 | 40.1 | 15.3 | 15.2 |
| 76 | 45.1 | 45.3 | 56.9 | 56 | 39.6 | 39.6 | 15.4 | 15.4 |
| 77 | 44.8 | 45.1 | 55.9 | 57.5 | 39.3 | 39.6 | 15.5 | 15.7 |
| 78 | 44.5 | 44.9 | 55.2 | 58 | 39.2 | 39.7 | 15.7 | 15.9 |
| 79 | 44.2 | 44.7 | 54.4 | 58.5 | 39 | 39.7 | 15.9 | 16.1 |
| 80 | 43.9 | 44.5 | 51.4 | 58.8 | 38.9 | 39.5 | 16.2 | 16.3 |
| 81 | 43.7 | 44.2 | 47.9 | 58.5 | 38.7 | 39.1 | 16.5 | 16.5 |
| 82 | 43.5 | 43.7 | 46 | 57.9 | 38.5 | 39 | 16.8 | 16.8 |
| 83 | 43.4 | 43 | 45.4 | 57.1 | 38.4 | 38.9 | 17 | 17 |
| 84 | 43.1 | 42.8 | 44.7 | 56.5 | 38.3 | 38.8 | 17.2 | 17.1 |
| 85 | 42.8 | 42.7 | 44 | 55.6 | 38.1 | 38.7 | 17.4 | 17.4 |
| 86 | 42.6 | 42.2 | 43.3 | 55.2 | 38 | 38.6 | 17.8 | 17.8 |
| 87 | 42.5 | 41.7 | 42.7 | 52.5 | 37.8 | 38.4 | 18.2 | 18.4 |
| 88 | 42.4 | 41.4 | 42.1 | 49 | 37.6 | 38.3 | 18.8 | 19 |
| 89 | 42 | 41.7 | 41.6 | 46.2 | 37.6 | 38.3 | 19.6 | 19.5 |
| 90 | 41.7 | 42.3 | 41 | 45.5 | 37.5 | 38.1 | 20.5 | 20.3 |
| 91 | 41.7 | 42.3 | 40.3 | 44.8 | 37.4 | 37.9 | 21.6 | 21.3 |
| 92 | 41.7 | 42.1 | 39.8 | 44 | 37.4 | 37.7 | 22.9 | 22.8 |
| 93 | 41.4 | 41.6 | 39.4 | 43.5 | 37.4 | 37.7 | 24.5 | 24.6 |
| 94 | 41.1 | 41.4 | 39.2 | 42.8 | 37.3 | 37.6 | 26.7 | 26.8 |
| 95 | 40.9 | 41.4 | 38.9 | 42.4 | 37.2 | 37.5 | 29.1 | 28.9 |
| 96 | 40.7 | 41.5 | 38.6 | 41.8 | 37 | 37.4 | 31.5 | 31.2 |
| 97 | 40.5 | 41.4 | 38.4 | 41.2 | 36.9 | 37.2 | 34.4 | 34.2 |
| 98 | 40.5 | 41.4 | 38.2 | 40.7 | 36.8 | 37.1 | 37.9 | 38 |
| 99 | 40.2 | 41.5 | 38 | 40.1 | 36.4 | 37 | 41.3 | 41.7 |
| 100 | 39.9 | 41.6 | 37.9 | 39.7 | 35.9 | 37 | 43.8 | 44.7 |
| 101 | 39.8 | 41.3 | 37.7 | 39.3 | 35.6 | 37 | 45.2 | 47.1 |
| 102 | 39.8 | 41.3 | 37.6 | 38.9 | 35.4 | 36.9 | 46 | 48.3 |
| 103 | 39.7 | 41.2 | 37.5 | 38.7 | 35.3 | 36.5 | 47 | 48.1 |
| 104 | 39.5 | 41.1 | 37.4 | 38.4 | 35.3 | 36.1 | 47.1 | 46.3 |
| 105 | 39.9 | 40.9 | 37.2 | 38.2 | 35.3 | 35.5 | 46.2 | 45.6 |
| 106 | 40.1 | 41.1 | 37.1 | 38 | 35.2 | 35.4 | 43 | 43.9 |
| 107 | 40 | 41.3 | 36.9 | 37.8 | 35.1 | 35.3 | 40.2 | 42.1 |
| 108 | 39.8 | 41.5 | 36.8 | 37.5 | 35.1 | 35.2 | 38.4 | 39 |
| 109 | 39.5 | 41.5 | 36.7 | 37.3 | 35 | 35.2 | 37.7 | 38 |
| 110 | 39.5 | 41.3 | 36.6 | 37.2 | 34.9 | 35.2 | 37.1 | 37.4 |
| 111 | 39.6 | 41.4 | 36.6 | 37 | 34.8 | 35.2 | 36.6 | 37.1 |
| 112 | 39.6 | 41.5 | 36.6 | 36.9 | 34.8 | 35.2 | 36.1 | 36.6 |
| 113 | 39.8 | 41.4 | 36.5 | 36.8 | 34.7 | 35 | 35.7 | 36.1 |
| 114 | 39.9 | 41.3 | 36.5 | 36.6 | 34.6 | 35 | 35.1 | 35.4 |
| 115 | 40 | 41.2 | 36.5 | 36.4 | 34.6 | 34.9 | 34.6 | 34.8 |
| 116 | 39.9 | 41.1 | 36.5 | 36.3 | 34.6 | 34.8 | 34 | 34.3 |
| 117 | 39.7 | 41 | 36.5 | 36.3 | 34.5 | 34.7 | 33.7 | 33.9 |
| 118 | 39.3 | 41 | 36.5 | 36.2 | 34.5 | 34.7 | 33.3 | 33.7 |
| 119 | 39.3 | 40.9 | 36.5 | 36.1 | 34.4 | 34.7 | 33.1 | 33.4 |
| 120 | 39.5 | 40.9 | 36.5 | 36 | 34.3 | 34.7 | 32.8 | 33.1 |
| 121 | 39.7 | 40.8 | 36.6 | 35.8 | 34.3 | 34.6 | 32.6 | 32.9 |
| 122 | 39.6 | 40.8 | 36.6 | 35.9 | 34.4 | 34.6 | 32.4 | 32.7 |
| 123 | 39.5 | 40.7 | 36.6 | 36 | 34.4 | 34.5 | 32.2 | 32.5 |
| 124 | 39.5 | 40.8 | 36.4 | 36 | 34.3 | 34.4 | 32 | 32.2 |
| 125 | 39.4 | 40.8 | 36.3 | 35.9 | 34.3 | 34.4 | 31.9 | 32 |
| 126 | 39.2 | 40.7 | 36.4 | 35.7 | 34.3 | 34.4 | 31.7 | 31.9 |
| 127 | 39 | 40.7 | 36.4 | 35.7 | 34.2 | 34.4 | 31.6 | 31.7 |
| 128 | 38.8 | 40.8 | 36.3 | 35.7 | 34.2 | 34.3 | 31.5 | 31.5 |
| 129 | 38.8 | 40.9 | 36.3 | 35.7 | 34.2 | 34.3 | 31.3 | 31.4 |
| 130 | 38.8 | 41 | 36.3 | 35.7 | 34.1 | 34.3 | 31.1 | 31.2 |
| 131 | 38.8 | 40.8 | 36.3 | 35.6 | 34.1 | 34.3 | 30.9 | 31.1 |
| 132 | 38.5 | 40.8 | 36.2 | 35.5 | 34 | 34.3 | 30.8 | 31 |
| 133 | 38.5 | 40.9 | 36.2 | 35.4 | 34 | 34.3 | 30.7 | 30.9 |
| 134 | 38.7 | 40.6 | 36.3 | 35.5 | 34.1 | 34.2 | 30.6 | 30.7 |
| 135 | 38.9 | 39.9 | 36.2 | 35.4 | 34.1 | 34.1 | 30.5 | 30.7 |
| 136 | 39 | 39.2 | 36.2 | 35.4 | 34.1 | 34 | 30.4 | 30.6 |
| 137 | 38.9 | 39.1 | 36.1 | 35.4 | 34.1 | 33.9 | 30.3 | 30.5 |
| 138 | 39 | 39.1 | 36.1 | 35.5 | 34.1 | 34 | 30.3 | 30.5 |
| 139 | 39.1 | 39.2 | 36 | 35.5 | 34.1 | 33.9 | 30.2 | 30.5 |
| 140 | 39 | 39 | 35.9 | 35.4 | 34 | 33.9 | 30.2 | 30.2 |
| 141 | 38.7 | 38.8 | 35.9 | 35.5 | 34 | 33.8 | 30.1 | 30.2 |
| 142 | 38.4 | 38.8 | 35.9 | 35.4 | 34 | 33.8 | 30 | 30.1 |
| 143 | 38.4 | 38.8 | 35.7 | 35.4 | 34 | 33.7 | 29.9 | 30 |
| 144 | 38.5 | 38.8 | 35.7 | 35.4 | 34 | 33.8 | 29.8 | 29.8 |
| 145 | 38.6 | 38.8 | 35.6 | 35.5 | 34 | 33.8 | 29.7 | 29.6 |
| 146 | 38.4 | 38.8 | 35.4 | 35.5 | 34 | 33.9 | 29.7 | 29.6 |
| 147 | 38.3 | 38.9 | 35.5 | 35.3 | 33.9 | 33.8 | 29.5 | 29.5 |
| 148 | 38.1 | 38.8 | 35.5 | 35.2 | 33.9 | 33.8 | 29.4 | 29.4 |
| 149 | 38.3 | 38.8 | 35.6 | 35.1 | 33.9 | 33.8 | 29.4 | 29.4 |
| 150 | 38.5 | 38.8 | 35.7 | 35 | 33.9 | 33.8 | 29.3 | 29.4 |
| 151 | 38.6 | 39 | 35.9 | 35 | 33.8 | 33.8 | 29.2 | 29.3 |
| 152 | 38.5 | 39.3 | 35.9 | 35 | 33.6 | 33.7 | 29.2 | 29.2 |
| 153 | 38.5 | 39.2 | 36 | 35 | 33.6 | 33.7 | 29.1 | 29.1 |
| 154 | 38.3 | 39 | 36.1 | 35 | 33.5 | 33.8 | 29 | 29.2 |
| 155 | 38.3 | 39.1 | 36.1 | 35 | 33.5 | 33.8 | 29 | 29.1 |
| 156 | 38.3 | 39.2 | 36 | 34.9 | 33.5 | 33.8 | 29 | 29 |
| 157 | 38.2 | 39.2 | 35.9 | 34.9 | 33.4 | 33.8 | 28.9 | 28.8 |
| 158 | 38.2 | 39 | 35.8 | 34.9 | 33.4 | 33.8 | 28.8 | 28.7 |
| 159 | 38.3 | 38.7 | 35.7 | 34.9 | 33.4 | 33.7 | 28.7 | 28.7 |
| 160 | 38.4 | 38.8 | 35.6 | 34.9 | 33.4 | 33.7 | 28.7 | 28.7 |
| 161 | 38.4 | 39 | 35.8 | 34.9 | 33.5 | 33.6 | 28.5 | 28.6 |
| 162 | 38 | 39 | 36 | 34.9 | 33.5 | 33.5 | 28.6 | 28.6 |
| 163 | 37.6 | 38.8 | 36 | 34.8 | 33.4 | 33.6 | 28.5 | 28.6 |
| 164 | 37.3 | 38.7 | 36.1 | 34.8 | 33.4 | 33.6 | 28.5 | 28.6 |
| 165 | 37.3 | 38.6 | 36 | 34.9 | 33.3 | 33.5 | 28.4 | 28.6 |
| 166 | 37.2 | 38.6 | 35.9 | 35.1 | 33.3 | 33.4 | 28.5 | 28.6 |
| 167 | 37.1 | 38.5 | 35.7 | 35.3 | 33.2 | 33.5 | 28.5 | 28.5 |
| 168 | 37.2 | 38.6 | 35.7 | 35.4 | 33.1 | 33.4 | 28.5 | 28.5 |
| 169 | 37.2 | 38.8 | 35.6 | 35.4 | 33 | 33.2 | 28.4 | 28.4 |
| 170 | 37.2 | 38.9 | 35.9 | 35.4 | 33 | 32.9 | 28.4 | 28.2 |
| 171 | 37.2 | 39.1 | 36 | 35.2 | 32.9 | 32.8 | 28.3 | 27.8 |
| 172 | 37.2 | 39.1 | 36.1 | 35 | 32.9 | 32.8 | 28.4 | 27.8 |
| 173 | 37.4 | 39.1 | 36.2 | 35 | 32.9 | 32.8 | 28.4 | 28.1 |
| 174 | 37.4 | 39.2 | 36.3 | 35 | 32.9 | 32.7 | 28.2 | 28.2 |
| 175 | 37.4 | 39.4 | 36.3 | 35 | 32.8 | 32.7 | 28.2 | 28.2 |
| 176 | 37.5 | 39.5 | 36.2 | 35 | 32.8 | 32.6 | 28.1 | 28.3 |
| 177 | 37.4 | 39.6 | 36.1 | 35.1 | 32.8 | 32.7 | 28 | 28.4 |
| 178 | 37.4 | 39.4 | 36 | 35.1 | 32.8 | 32.7 | 27.8 | 28.3 |
| 179 | 37.4 | 39.1 | 35.9 | 35.1 | 32.8 | 32.6 | 27.6 | 28.2 |
| 180 | 37.3 | 38.7 | 35.9 | 35.1 | 32.8 | 32.6 | 27.7 | 28.3 |
| 181 | 37.2 | 38.5 | 36.1 | 35.2 | 32.7 | 32.5 | 28 | 28.3 |
| 182 | 37.2 | 38.5 | 36.1 | 35.3 | 32.7 | 32.4 | 28 | 28.5 |
| 183 | 37.2 | 38.5 | 36 | 35.3 | 32.6 | 32.3 | 27.9 | 28.5 |
| 184 | 37.2 | 38.5 | 35.8 | 35.3 | 32.5 | 32.3 | 27.9 | 28.4 |
| 185 | 37.2 | 38.5 | 35.7 | 35.2 | 32.5 | 32.3 | 28 | 28.4 |
| 186 | 37.2 | 38.6 | 35.5 | 35.2 | 32.5 | 32.3 | 28 | 28.1 |
| 187 | 37.2 | 38.6 | 35.4 | 35.3 | 32.5 | 32.3 | 28 | 27.7 |
| 188 | 37.2 | 38.6 | 35.3 | 35.4 | 32.5 | 32.2 | 28.1 | 27.5 |
| 189 | 37.2 | 38.7 | 35.4 | 35.4 | 32.4 | 32.2 | 28 | 27.5 |
| 190 | 37.3 | 38.8 | 35.7 | 35.3 | 32.4 | 32.2 | 27.9 | 27.5 |
| 191 | 37.3 | 38.7 | 35.7 | 35.3 | 32.4 | 32.1 | 27.7 | 27.4 |
| 192 | 37.3 | 38.7 | 35.6 | 35.2 | 32.4 | 32 | 27.4 | 27.4 |
| 193 | 37.2 | 38.7 | 35.5 | 35.1 | 32.4 | 32 | 27.4 | 27.4 |
| 194 | 37.2 | 38.6 | 35.5 | 35 | 32.3 | 31.9 | 27.4 | 27.4 |
| 195 | 37.1 | 38.7 | 35.7 | 34.8 | 32.3 | 31.9 | 27.5 | 27.4 |
| 196 | 37.1 | 38.7 | 36 | 34.5 | 32.2 | 31.9 | 27.5 | 27.6 |
| 197 | 37.2 | 38.6 | 36 | 34.2 | 32.1 | 31.9 | 27.4 | 28 |
| 198 | 37.3 | 38.6 | 35.9 | 34.1 | 32.1 | 31.8 | 27.3 | 28 |
| 199 | 37.3 | 38.7 | 35.8 | 34.2 | 32.1 | 31.8 | 27.3 | 27.6 |

**Figure S4.** Pasting profiles of the refined tritordeum (gray dotted line) and bread wheat (gray line) flours, and of the semi-whole tritordeum (dotted black line) and bread wheat (black line) flours obtained from the Micro Visco-Amylo-Graph test.

Temperature (°C)

Torque (BU)

| Time (min) | Temperature | Bread wheat,refined flour | Bread wheat, semi-whole flour | Tritordeum, refined flour | Tritordeum, semi-whole flour |
| --- | --- | --- | --- | --- | --- |
| 0 | 30 | 6 | 6 | 5 | 5 |
| 0.0333333 | 30.1 | 6 | 5 | 5 | 5 |
| 0.0666667 | 30.2 | 6 | 5 | 5 | 5 |
| 0.1 | 30.3 | 6 | 5 | 5 | 5 |
| 0.133333 | 30.4 | 6 | 5 | 5 | 5 |
| 0.166667 | 30.5 | 6 | 5 | 5 | 5 |
| 0.2 | 30.6 | 6 | 5 | 5 | 5 |
| 0.233333 | 30.7 | 6 | 5 | 5 | 5 |
| 0.266667 | 30.8 | 6 | 5 | 5 | 5 |
| 0.3 | 30.9 | 6 | 5 | 5 | 5 |
| 0.333333 | 31 | 6 | 5 | 5 | 5 |
| 0.366667 | 31.1 | 6 | 5 | 5 | 5 |
| 0.4 | 31.2 | 6 | 5 | 5 | 5 |
| 0.433333 | 31.3 | 6 | 5 | 5 | 5 |
| 0.466667 | 31.4 | 6 | 5 | 5 | 5 |
| 0.5 | 31.5 | 6 | 5 | 5 | 5 |
| 0.533333 | 31.6 | 6 | 5 | 5 | 5 |
| 0.566667 | 31.7 | 6 | 5 | 5 | 5 |
| 0.6 | 31.8 | 6 | 5 | 5 | 5 |
| 0.633333 | 31.9 | 6 | 5 | 5 | 5 |
| 0.666667 | 32 | 6 | 5 | 5 | 5 |
| 0.7 | 32.1 | 6 | 5 | 5 | 5 |
| 0.733333 | 32.2 | 6 | 5 | 5 | 5 |
| 0.766667 | 32.3 | 6 | 5 | 5 | 5 |
| 0.8 | 32.4 | 6 | 5 | 5 | 5 |
| 0.833333 | 32.5 | 6 | 5 | 5 | 5 |
| 0.866667 | 32.6 | 6 | 5 | 5 | 5 |
| 0.9 | 32.7 | 6 | 5 | 5 | 5 |
| 0.933333 | 32.8 | 6 | 5 | 5 | 5 |
| 0.966667 | 32.9 | 6 | 5 | 5 | 5 |
| 1 | 33 | 6 | 5 | 5 | 5 |
| 1.03333 | 33.1 | 6 | 5 | 5 | 5 |
| 1.06667 | 33.2 | 6 | 5 | 5 | 5 |
| 1.1 | 33.3 | 6 | 5 | 5 | 5 |
| 1.13333 | 33.4 | 5 | 5 | 5 | 5 |
| 1.16667 | 33.5 | 6 | 5 | 5 | 5 |
| 1.2 | 33.6 | 6 | 5 | 5 | 5 |
| 1.23333 | 33.7 | 6 | 5 | 5 | 5 |
| 1.26667 | 33.8 | 6 | 5 | 5 | 5 |
| 1.3 | 33.9 | 5 | 5 | 5 | 5 |
| 1.33333 | 34 | 6 | 5 | 5 | 5 |
| 1.36667 | 34.1 | 6 | 5 | 5 | 5 |
| 1.4 | 34.2 | 6 | 5 | 5 | 5 |
| 1.43333 | 34.3 | 6 | 5 | 5 | 5 |
| 1.46667 | 34.4 | 5 | 5 | 5 | 5 |
| 1.5 | 34.5 | 5 | 5 | 5 | 5 |
| 1.53333 | 34.6 | 5 | 5 | 5 | 5 |
| 1.56667 | 34.7 | 5 | 5 | 5 | 5 |
| 1.6 | 34.8 | 5 | 5 | 4 | 5 |
| 1.63333 | 34.9 | 5 | 5 | 4 | 5 |
| 1.66667 | 35 | 5 | 5 | 4 | 5 |
| 1.7 | 35.1 | 5 | 5 | 4 | 4 |
| 1.73333 | 35.2 | 5 | 5 | 4 | 5 |
| 1.76667 | 35.3 | 5 | 5 | 4 | 5 |
| 1.8 | 35.4 | 5 | 5 | 4 | 5 |
| 1.83333 | 35.5 | 5 | 5 | 4 | 5 |
| 1.86667 | 35.6 | 5 | 5 | 4 | 5 |
| 1.9 | 35.7 | 5 | 5 | 4 | 4 |
| 1.93333 | 35.8 | 5 | 5 | 4 | 4 |
| 1.96667 | 35.9 | 5 | 5 | 4 | 5 |
| 2 | 36 | 5 | 5 | 4 | 4 |
| 2.03333 | 36.1 | 5 | 5 | 4 | 5 |
| 2.06667 | 36.2 | 5 | 5 | 4 | 4 |
| 2.1 | 36.3 | 5 | 5 | 4 | 4 |
| 2.13333 | 36.4 | 5 | 5 | 4 | 4 |
| 2.16667 | 36.5 | 5 | 5 | 4 | 4 |
| 2.2 | 36.6 | 5 | 5 | 4 | 4 |
| 2.23333 | 36.7 | 5 | 5 | 4 | 5 |
| 2.26667 | 36.8 | 5 | 5 | 4 | 4 |
| 2.3 | 36.9 | 5 | 5 | 4 | 4 |
| 2.33333 | 37 | 5 | 5 | 4 | 4 |
| 2.36667 | 37.1 | 5 | 5 | 4 | 4 |
| 2.4 | 37.2 | 5 | 5 | 4 | 4 |
| 2.43333 | 37.3 | 5 | 5 | 4 | 4 |
| 2.46667 | 37.4 | 5 | 5 | 4 | 4 |
| 2.5 | 37.5 | 5 | 5 | 4 | 4 |
| 2.53333 | 37.6 | 5 | 5 | 4 | 4 |
| 2.56667 | 37.7 | 5 | 5 | 4 | 4 |
| 2.6 | 37.8 | 5 | 5 | 4 | 4 |
| 2.63333 | 37.9 | 5 | 4 | 4 | 4 |
| 2.66667 | 38 | 5 | 5 | 4 | 4 |
| 2.7 | 38.1 | 5 | 5 | 4 | 4 |
| 2.73333 | 38.2 | 5 | 5 | 4 | 4 |
| 2.76667 | 38.3 | 5 | 4 | 4 | 4 |
| 2.8 | 38.4 | 5 | 5 | 4 | 4 |
| 2.83333 | 38.5 | 5 | 4 | 4 | 4 |
| 2.86667 | 38.6 | 5 | 5 | 4 | 4 |
| 2.9 | 38.7 | 5 | 4 | 4 | 4 |
| 2.93333 | 38.8 | 5 | 4 | 4 | 4 |
| 2.96667 | 38.9 | 5 | 4 | 4 | 4 |
| 3 | 39 | 5 | 4 | 4 | 4 |
| 3.03333 | 39.1 | 5 | 4 | 4 | 4 |
| 3.06667 | 39.2 | 5 | 4 | 4 | 4 |
| 3.1 | 39.3 | 5 | 4 | 4 | 4 |
| 3.13333 | 39.4 | 5 | 4 | 4 | 4 |
| 3.16667 | 39.5 | 5 | 4 | 4 | 4 |
| 3.2 | 39.6 | 5 | 4 | 4 | 4 |
| 3.23333 | 39.7 | 5 | 4 | 4 | 4 |
| 3.26667 | 39.8 | 5 | 4 | 4 | 4 |
| 3.3 | 39.9 | 5 | 4 | 4 | 4 |
| 3.33333 | 40 | 5 | 4 | 4 | 4 |
| 3.36667 | 40.1 | 5 | 4 | 4 | 4 |
| 3.4 | 40.2 | 5 | 4 | 4 | 4 |
| 3.43333 | 40.3 | 5 | 4 | 4 | 4 |
| 3.46667 | 40.4 | 5 | 4 | 4 | 4 |
| 3.5 | 40.5 | 5 | 4 | 4 | 4 |
| 3.53333 | 40.6 | 5 | 4 | 4 | 4 |
| 3.56667 | 40.7 | 5 | 4 | 4 | 4 |
| 3.6 | 40.8 | 5 | 4 | 4 | 4 |
| 3.63333 | 40.9 | 5 | 4 | 4 | 4 |
| 3.66667 | 41 | 5 | 4 | 4 | 4 |
| 3.7 | 41.1 | 5 | 4 | 4 | 4 |
| 3.73333 | 41.2 | 5 | 4 | 4 | 4 |
| 3.76667 | 41.3 | 5 | 4 | 4 | 4 |
| 3.8 | 41.4 | 5 | 4 | 4 | 4 |
| 3.83333 | 41.5 | 5 | 4 | 4 | 4 |
| 3.86667 | 41.6 | 5 | 4 | 4 | 4 |
| 3.9 | 41.7 | 5 | 4 | 4 | 4 |
| 3.93333 | 41.8 | 5 | 4 | 4 | 4 |
| 3.96667 | 41.9 | 5 | 4 | 4 | 4 |
| 4 | 42 | 5 | 4 | 4 | 4 |
| 4.03333 | 42.1 | 5 | 4 | 4 | 4 |
| 4.06667 | 42.2 | 5 | 4 | 4 | 4 |
| 4.1 | 42.3 | 5 | 4 | 4 | 4 |
| 4.13333 | 42.4 | 5 | 4 | 4 | 4 |
| 4.16667 | 42.5 | 5 | 4 | 4 | 4 |
| 4.2 | 42.6 | 5 | 4 | 4 | 4 |
| 4.23333 | 42.7 | 5 | 4 | 4 | 4 |
| 4.26667 | 42.8 | 5 | 4 | 4 | 4 |
| 4.3 | 42.9 | 5 | 4 | 4 | 4 |
| 4.33333 | 43 | 5 | 4 | 4 | 4 |
| 4.36667 | 43.1 | 5 | 4 | 4 | 4 |
| 4.4 | 43.2 | 5 | 4 | 4 | 4 |
| 4.43333 | 43.3 | 5 | 4 | 4 | 4 |
| 4.46667 | 43.4 | 5 | 4 | 4 | 4 |
| 4.5 | 43.5 | 5 | 4 | 4 | 4 |
| 4.53333 | 43.6 | 5 | 4 | 4 | 4 |
| 4.56667 | 43.7 | 5 | 4 | 4 | 4 |
| 4.6 | 43.8 | 5 | 4 | 4 | 4 |
| 4.63333 | 43.9 | 5 | 4 | 4 | 4 |
| 4.66667 | 44 | 5 | 4 | 4 | 4 |
| 4.7 | 44.1 | 5 | 4 | 4 | 4 |
| 4.73333 | 44.2 | 5 | 4 | 4 | 4 |
| 4.76667 | 44.3 | 5 | 4 | 4 | 4 |
| 4.8 | 44.4 | 5 | 4 | 4 | 4 |
| 4.83333 | 44.5 | 5 | 4 | 4 | 4 |
| 4.86667 | 44.6 | 5 | 4 | 4 | 4 |
| 4.9 | 44.7 | 5 | 4 | 3 | 4 |
| 4.93333 | 44.8 | 5 | 4 | 4 | 4 |
| 4.96667 | 44.9 | 5 | 4 | 4 | 4 |
| 5 | 45 | 5 | 4 | 4 | 4 |
| 5.03333 | 45.1 | 5 | 4 | 3 | 4 |
| 5.06667 | 45.2 | 5 | 4 | 3 | 4 |
| 5.1 | 45.3 | 5 | 4 | 3 | 4 |
| 5.13333 | 45.4 | 5 | 4 | 3 | 4 |
| 5.16667 | 45.5 | 5 | 4 | 3 | 4 |
| 5.2 | 45.6 | 5 | 4 | 3 | 4 |
| 5.23333 | 45.7 | 5 | 4 | 3 | 4 |
| 5.26667 | 45.8 | 5 | 4 | 3 | 4 |
| 5.3 | 45.9 | 5 | 4 | 3 | 4 |
| 5.33333 | 46 | 5 | 4 | 3 | 4 |
| 5.36667 | 46.1 | 5 | 4 | 3 | 4 |
| 5.4 | 46.2 | 5 | 4 | 3 | 4 |
| 5.43333 | 46.3 | 5 | 4 | 3 | 4 |
| 5.46667 | 46.4 | 5 | 4 | 3 | 4 |
| 5.5 | 46.5 | 5 | 4 | 3 | 4 |
| 5.53333 | 46.6 | 5 | 4 | 3 | 4 |
| 5.56667 | 46.7 | 5 | 4 | 3 | 4 |
| 5.6 | 46.8 | 5 | 4 | 3 | 3 |
| 5.63333 | 46.9 | 5 | 4 | 3 | 4 |
| 5.66667 | 47 | 5 | 4 | 3 | 4 |
| 5.7 | 47.1 | 5 | 4 | 3 | 4 |
| 5.73333 | 47.2 | 5 | 4 | 3 | 4 |
| 5.76667 | 47.3 | 5 | 4 | 6 | 4 |
| 5.8 | 47.4 | 5 | 4 | 4 | 4 |
| 5.83333 | 47.5 | 5 | 4 | 3 | 4 |
| 5.86667 | 47.6 | 5 | 4 | 3 | 3 |
| 5.9 | 47.7 | 5 | 4 | 3 | 4 |
| 5.93333 | 47.8 | 5 | 4 | 3 | 4 |
| 5.96667 | 47.9 | 5 | 4 | 3 | 3 |
| 6 | 48 | 5 | 4 | 3 | 3 |
| 6.03333 | 48.1 | 5 | 4 | 3 | 3 |
| 6.06667 | 48.2 | 5 | 4 | 3 | 4 |
| 6.1 | 48.3 | 5 | 4 | 3 | 3 |
| 6.13333 | 48.4 | 5 | 4 | 3 | 3 |
| 6.16667 | 48.5 | 5 | 4 | 3 | 3 |
| 6.2 | 48.6 | 5 | 4 | 3 | 3 |
| 6.23333 | 48.7 | 5 | 4 | 3 | 3 |
| 6.26667 | 48.8 | 5 | 4 | 3 | 3 |
| 6.3 | 48.9 | 5 | 4 | 3 | 3 |
| 6.33333 | 49 | 5 | 4 | 3 | 4 |
| 6.36667 | 49.1 | 5 | 3 | 3 | 4 |
| 6.4 | 49.2 | 5 | 4 | 3 | 3 |
| 6.43333 | 49.3 | 5 | 4 | 3 | 3 |
| 6.46667 | 49.4 | 5 | 4 | 3 | 3 |
| 6.5 | 49.5 | 5 | 4 | 3 | 3 |
| 6.53333 | 49.6 | 5 | 4 | 3 | 4 |
| 6.56667 | 49.7 | 5 | 4 | 3 | 3 |
| 6.6 | 49.8 | 5 | 3 | 3 | 3 |
| 6.63333 | 49.9 | 5 | 4 | 3 | 3 |
